# Stochastic Simulation of Endemic *Salmonella enterica* Serovar Typhi: The Importance of Long Lasting Immunity and the Carrier State

**DOI:** 10.1371/journal.pone.0074097

**Published:** 2013-09-10

**Authors:** Allan Saul, Tom Smith, Nicolas Maire

**Affiliations:** 1 Novartis Vaccines Institute for Global Health, Siena, Italy; 2 Swiss Tropical and Public Health Institute, Basel, Switzerland; 3 University of Basel, Basel, Switzerland; University of Illinois at Urbana-Champaign, United States of America

## Abstract

**Background:**

Typhoid fever caused by *Salmonella enterica* serovar Typhi (*S*. Typhi) remains a serious burden of disease, especially in developing countries of Asia and Africa. It is estimated that it causes 200,000 deaths per year, mainly in children. *S.* Typhi is an obligate pathogen of humans and although it has a relatively complex life cycle with a long lived carrier state, the absence of non-human hosts suggests that well targeted control methods should have a major impact on disease. Newer control methods including new generations of vaccines offer hope but their implementation would benefit from quantitative models to guide the most cost effective strategies. This paper presents a quantitative model of Typhoid disease, immunity and transmission as a first step in that process.

**Methodology/Principal Findings:**

A stochastic agent-based model has been developed that incorporates known features of the biology of typhoid including probability of infection, the consequences of infection, treatment options, acquisition and loss of immunity as a result of infection and vaccination, the development of the carrier state and the impact of environmental or behavioral factors on transmission. The model has been parameterized with values derived where possible from the literature and where this was not possible, feasible parameters space has been determined by sensitivity analyses, fitting the simulations to age distribution of field data. The model is able to adequately predict the age distribution of typhoid in two settings.

**Conclusions/Significance:**

The modeling highlights the importance of variations in the exposure/resistance of infants and young children to infection in different settings, especially as this impacts on design of control programs; it predicts that naturally induced clinical and sterile immunity to typhoid is long lived and highlights the importance of the carrier state especially in areas of low transmission.

## Introduction

*Salmonella enterica* serovar Typhi (Typhi), the causal agent of typhoid, is a bacterial pathogen transmitted between humans, by ingestion of contaminated faces and urine. In many low-income settings *S.* Typhi is endemic, but elsewhere sporadic epidemics occur. It was a major cause of death in the industrialized world until the early 20^th^ century, when disease rates fell considerably due to improved sanitation. Nevertheless, despite further substantial advances partly as a result of effective antibiotic therapy, the global burden remains substantial.

Immunity to Typhi is poorly understood. Both natural exposure and live attenuated vaccines provide some protection, but volunteer infection studies [1] and naturally occurring epidemics [2] have both demonstrated repeated infection with symptomatic typhoid. A specific element of the biology is the existence of long-term carrier states involving colonization of the gall bladder and of other sites. The ways in which all these factors interact to determine level of transmission in particular environments is complex. In particular in endemic areas, the reservoir of carriers, the continuing supply of susceptibles (new born or migrants) and the impact of immunity may all play a role in determining the incidence of disease and the transition between epidemic and stable endemic typhoid disease.

Live attenuated vaccines and polysaccharide vaccines targeting the Vi capsular antigen have limited efficacy and duration of protection. While vaccination is recommended by WHO for campaign vaccination of school age children [3], uptake has been limited. The clinical development of a conjugate vaccine [4]–[6], expected to have higher efficacy and to protect for longer and to be effective in infants, highlights the need for quantitative understanding of the dynamics of transmission and immunity.

Computer models of typhoid transmission provide a means of exploring the importance of different parameters, to test our understanding of the biology and to plan intervention strategies. Two models of typhoid transmission have been published [7], [8]. Both of these are deterministic models. This paper describes an agent based stochastic simulation model of the population dynamics of Typhi. This approach was used since an agent based model allows a direct translation of the qualitative description of the biology and their assumptions into the model; allows for simpler procedures for encoding events occurring at discrete intervals or for fixed times (e.g. an infectious period of 30 days rather than a population whose infectiousness decays exponentially with a half live of 30 days); allows for simpler encoding of treatment procedures by specifying which individuals are treated (e.g. 80% of children are vaccinated on their second birthday and of those previously vaccinated, 50% will be re-vaccinated 3 years later). This approach also allows simpler encoding of stochastic processes (e.g. what is the likely range of incidence rates following a certain vaccination program; what is the probability that the typhoid will be eliminated from a community of defined size). On the other hand this procedure generates large datasets that require more complex post model analysis, may require more computing power and has added complexity in fitting the model to data to estimate model parameters.

This model is calibrated using available field data. In this paper, it is used to explore the impact of altering transmission levels, of the acquisition and loss of clinical and sterile immunity and the importance of the carrier state on the observed age specific incidence of typhoid and on the stability of typhoid transmission in the community. The basic model has been designed to allow extension for modeling interventions such as vaccines, changed environmental or health delivery practices and modeling of epidemics but these applications will be described elsewhere.

## Model

The model is a discrete time stochastic simulation of a human population exposed to Typhi. Implementation of this creates an *in silico* population with age, gender, immune status (natural and vaccine), time since infection or vaccination and carrier status tracked individually. It comprises distinct modules simulating the demography, infection status, and immune status of the host population, as described below:

### Human Demography

The population model requires the following parameters which remain fixed throughout the simulation.

1. Crude birth rate. This is expressed as the number of births per 1000 total population per annum.
2. The starting population size.
3. The age specific mortality rates in the absence of typhoid infections. These define a life-table which is used to specify the age structure of the population

The population is initialized by assigning individuals randomly to age and gender to achieve a starting population with the desired age structure and with individuals having an equal probability of being male or female. Individuals may be assigned into distinct risk groups varying in behaviors and environmental factors related to typhoid.

In-migration rates may be specified, with immigrants characterized by age, gender, infection and immune status, with rates specified relative to the size of the existing population. The actual number of migrants and their status (age, gender, immunity) are assigned each month using random number generators. The start and end date of migrations can be specified. The migration module can be used to simulate a number of situations, e.g. to introduce a continuing source of naive individuals into a highly endemic area or to introduce a source of infection into a naive community to start an outbreak.

Births and non-typhoid deaths are simulated from Poisson processes with the predetermined birth, death and migration rates. The model has been extensively run using birth rates and age specific mortality from a DSS study in Matlab Bangladesh covering the period 1999 to 2002 [9] (assuming zero migration and no typhoid). The birth rates have been falling in Bangladesh over the period 1950 to 2002 and the death rates declining however the population growth rate has been steady over this period at approximately 2%. The initial age distribution derived from the life table approximates that independently observed in this Bangladesh population [10] This generates a demographically stable state in the virtual population, with an annual growth rate of 2%, defines the standard population used to initialize the simulations reported here. Typhoid mortality is additional to the mortality input to the simulation and leads to changes in the age structure over time. This model implicitly assumes that the target population is growing, but has a stable age structure. For 2000, model predicts an age distribution that approximates the UN population division estimates in Bangladesh for the percentage of both sexes by age group (Table 1) for most ages, except for the oldest age groups, presumably because the age specific mortality estimated from 1999–2002 underestimates the historical age specific mortalities for these age groups. As detailed below, the age distribution of typhoid fever in the community suggests that duration of immunity is very long lived. Hence the time taken to obtain stable level endemic typhoid fever will also be long, of the order of a human lifetime. In populations with high incidence of typhoid fever in places such as Dhaka and Kolkata, the age structure of the population may not be stable over these time periods. This is a potential limitation of the model. Modeling situations with a fluctuating age distribution is beyond the scope of this study.

**Table 1:** Estimated and modelled population distributions for the Bangladesh population for 2000.

Age group	UN Population estimate	Model prediction
0–4	12.6	13.3
5–9	12.4	11.6
10–14	12.0	10.6
15–19	11.1	9.3
20–39	31.7	28.0
40–59	14	17.2
60+	6.2	9.9

### Infection

Simulated infections are introduced into the population once the demography is in a stable state. With a single introduction an epidemic occurs that may or may not become endemic. As detailed below, to force a stable endemic state, multiple introductions can be made over a period of time.

**Figure 1:**
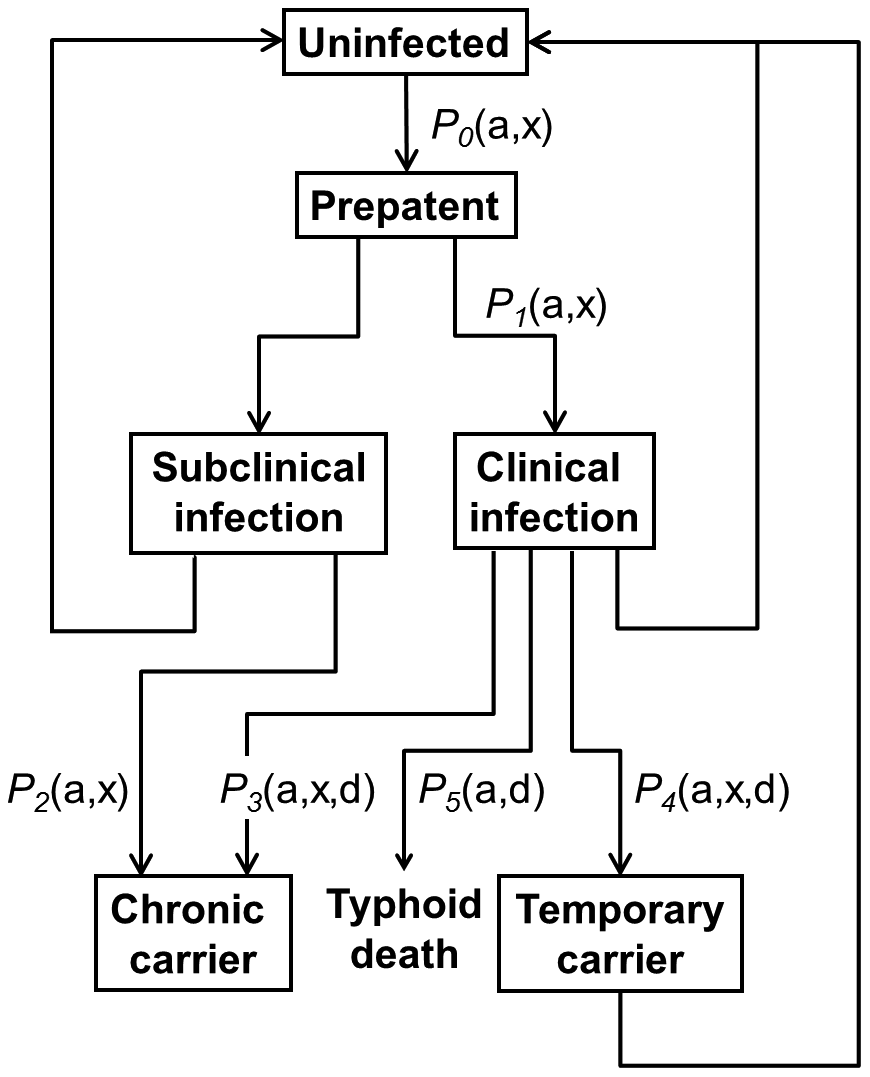
Infection states. The infection states and their transitions for the simplest model case (single homogeneous population with a single strain of *S*. Typhi). Uninfected individuals have a *P_0_* probability of becoming infected during each time step and thus time spent in this state depends on the force of infection and their immune status. For all other states, the time spent is sampled from a defined distribution. Where there are multiple possible transitions out of a state, conditional probabilities determine which path an individual follows. The order in which the probabilities are applied is specified in the text. Probabilities for each individual may depend on a: age; x: previous exposure/immune status; or d: treatment options and to a single time step.

Fig. 1 lists the allowed infection states within the model and the possible transitions between them, while Table 2 lists the functions determining the duration of infections and infectiousness. Each period of infection (indexed by k) is assigned a single value for its infectiousness sampled from a distribution that depends on the age of the host at the time of acquisition of the infection, 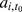, and the risk group of the host, 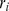. Some of the sojourn times within these states are fixed quantities, while others are stochastic (Table 2).

**Table 2:** Infection states.

State	State indicator	Duration of state(in the absence of death)	Infectiousness(infections per unit time)
Uninfected	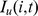	–	0
Prepatent	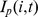	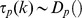	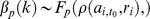
Subclinical	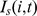	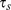	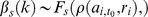
Acute clinical	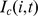	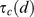	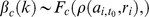
Temporary carrier	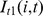	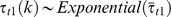	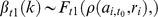
Chronic carrier	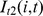	∞	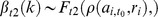

Any proper distributions can be used for the infectiousness values, 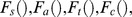 and for the durations of the pre-patent period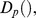 providing these are all constrained to be positive. In general, acute clinical infections are less infective than pre-patent or subclinical infections, i.e. 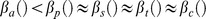. The quantity 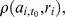 is a scale factor dependent on the age group at the start of the infection, 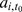, and risk group, 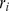, of simulated individual *i*. 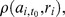
multiplies the infectiousness for any infection state and quantifies environmental influences on the contribution to the infectious reservoir. The exposure of individual *i* is also dependent on age and risk-group specific factors, which may vary over time (in particular by season) and are quantified by scale factor 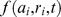. The expected value of the exposure is thus the product:
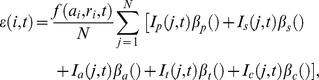
where infectiousness terms refer to the infections present in the *N* members of the community at time *t*.

The force of infection at any one time, *t*, for individual *i*,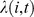 is then the product of the exposure and the susceptibility, which in the absence of refractoriness or immunity is 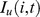, i.e.:
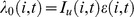



This model uses a discrete time implementation with time step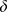. New infections are introduced via a Bernoulli process with probability of infection at any one time step:
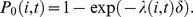



After infection, simulated individuals progress through the decision tree shown in Fig. 1, remaining in each state for the sojourn times defined in Table 2, and transitioning to new states with probabilities 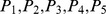 as shown in Fig. 1.

The initial period of all infections is assigned to a pre-patent state of short duration, after which the host becomes a clinical case with probability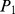. Infections that do not result in clinical disease continue with a further period of sub-clinical infection. The probability that person is infected each time step depends on the force of infection and the immune status. A person with sterile infection or vaccine induced immunity has a zero probability of becoming infected. This model uses the same probability of infection for both a fully susceptible person (leading to either a clinical or sub-clinical infection) or for a person with natural or vaccine induced clinical immunity (leading only to a sub-clinical infection). Sub-clinical infections are assigned a duration, 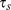, beyond the initial pre-patent period. The duration of clinical infections, 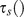 depends on how they are treated. Details of the case management model are given below. At the end of the acute phase there are three outcomes (Fig. 1): (1) The infection is cured (either naturally or following drug treatment); (2) the person becomes a chronically infected carrier and it is assumed that without treatment that this carrier state lasts a lifetime; or (3) the person becomes a temporarily infected carrier undergoing a series of relapses and that without further treatment, will resolve.

This model does not allow for superinfection: a person cannot be simultaneously infected with more than one strain. Temporary or chronic carriers are also assumed to be refractory to further infection. In this paper, only simulations with a single strain of Typhi are shown. The current version of the model allows for two strains that differ in their transmissibility, drug sensitivity and infection transition probabilities.

### Refractoriness and Immunity

Five different types of refractoriness and immunity may modify the susceptibility of a simulated individual and the outcome of infection (Table 3).

**Table 3:** Immunity and refractoriness.

State	Stateindicator	Duration(in absence of death)	Durationmodulated by	Stimulatingevent	Probabilityinduced$	Effect on newinfections
Infant Refractory/non-exposure	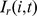	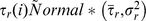	No modulation	Birth	1	Completelyprevented
Non-immune	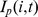	Until next infection	Next infection	Any type ofinfection	1	No effect
Infection-inducedsterile immunity	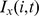	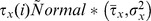	No modulation	Any type ofinfection	*P*_6_, (*P*_9_)&, (*P*_8_)^#&^	Completelyprevented
Infection-inducedclinical immunity	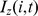	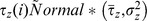	No modulation	Any type ofinfection	*P*_7_, (*P*_10_)&	New infectionsare sub-clinical
Infected	See Table 2	Dependent on type ofinfection (Table 2)	See Table 2	Infection	1	No superinfectionpossible

**Figure 2:**
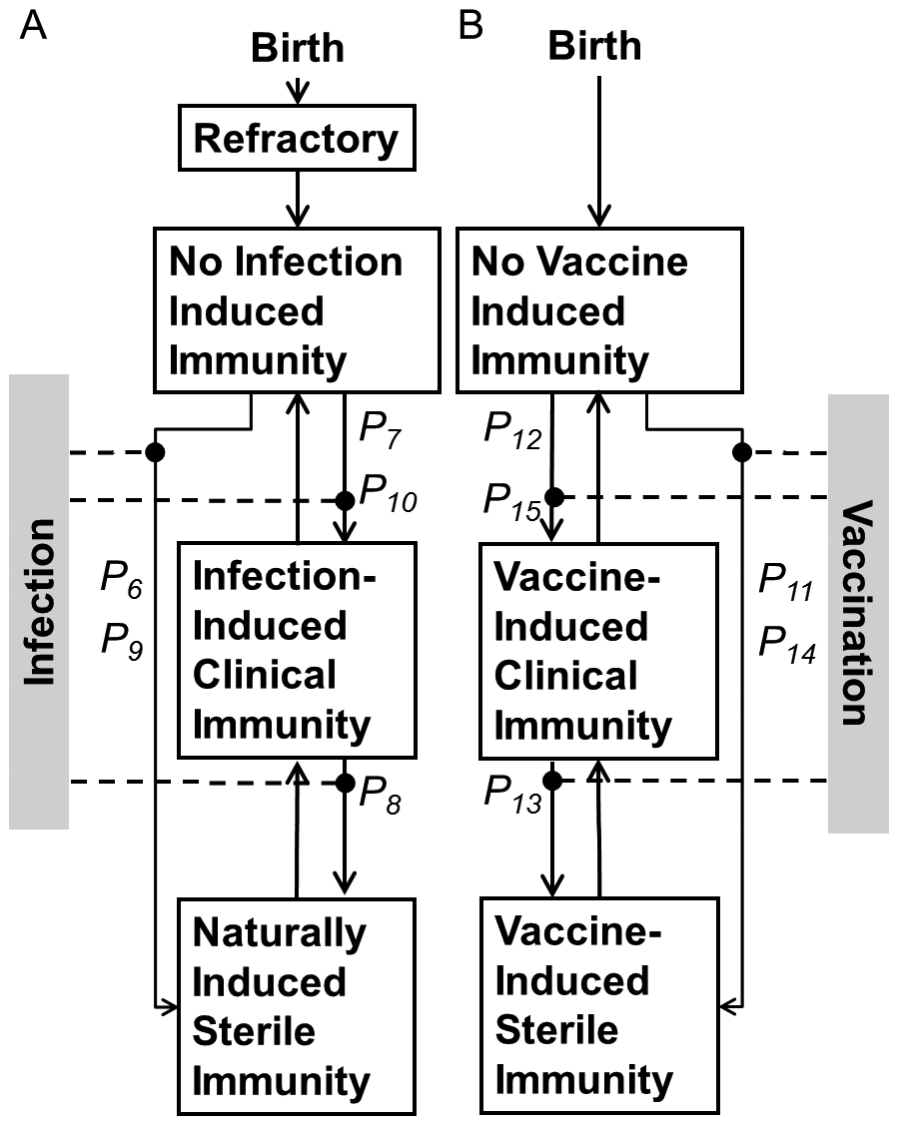
Immune states. The immune states and their transitions for infection and vaccine induced immunity. For individuals in the non-immune categories the time in this category depends on the infection rates and vaccination strategies. For other immune states, time in the state before losing immunity is sampled from a defined distribution, modulated by exposure/vaccination history. Where there are multiple possible transitions out of a state, conditional probabilities determine which path an individual follows. The order in which the probabilities are applied is specified in the text. *P_6_*, *P_7_*, *P_8_* refer to subclinical infections, *P_9_*, *P_10_* to clinical infections. *P_11_, P_12_* probabilities of inducing immunity in non-vaccinated individuals, *P_13_, P_14_, P_15_* in previously vaccinated individuals.

$ This is the probability that an eligible host exposed to the stimulus acquires this state (Figure 2).

* These durations are independently sampled from normal distributions. If the sampled duration is negative then the duration implemented is zero.

& These probabilities of acquisition apply to sub-clinical infections (Figure 2).

# These probabilities of acquisition apply to clinically-immune hosts (Figure 2).

Both infections and vaccines can lead to sterile immunity or clinical immunity. On exposure to Typhi, A person with sterile immunity is unable to be infected at all and this exposure has no effect on immunity. By contrast, on exposure to Typhi, a person with clinical immunity may become sub-clinically infected, this may change the person’s level of infection induced immunity and the subclinical infection will result in Typhi being released into the environment. Although a clinically immune person may be sub-clinically infected, this person will not develop a clinical infection. Although sub-clinical infections are common in experimental challenge [11] and the clinically immune state has been widely postulated (e.g. as discussed in Hornick et al., [11]) there is little direct evidence of immune states leading to “clinical immunity” and in many field situations e.g. vaccine trials it would be difficult to distinguish the two states. In this model, since the probability and duration of both clinical and sterile immunity are defined independently, the model can be run under conditions of no clinical or no sterile or a mixture of clinical and sterile immunity.

Corresponding to each of these types a state indicator takes a value of 1 when the individual is refractory or immune, and 0 when this is not the case. Each type of immunity or refractoriness is characterized by an effect on new infections, and also by a stochastically determined duration, which is sampled from a Normal distribution (Table 3). All immune effects including vaccine-induced immunity are treated as all-or-none phenomenon for the immune individual. I.e. there is no allowance for “leaky” immunity. For example, a vaccine that induces 70% immunity completely protects 70% of the population and offers no protection to the other 30%. While this is almost certainly a simplification, it is consistent with the wide range of immune responses seen in vaccination trials [5], and the association between antibody levels and protection from typhoid both suggest that within a population there will be “protected” and “nonprotected” individuals [12]. Therefore, it follows that at any given time, individuals are either completely susceptible, and hence experience force of infection 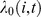 or are refractory. Allowing for immunity, the force of infection for individual i at time t is thus:
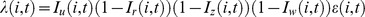



### Transitions between Immune States

Each simulated individual can transition between immune states with probabilities indicated in Fig. 2.

All new born children are initially assigned to an uninfectible class. This reflects the observation from highly endemic areas that there is usually a markedly lower incidence of typhoid fever in very young infants (e.g. <12 months) in hospital [13] and community based studies [14]. This has not been an invariant finding [15] and vertical transmission to neonates from carrier mothers have been reported [16], [17] so in principle, neonates in at least some populations are susceptible. The period of this lower incidence appears highly variable between different sites. For example, the peak incidence of typhoid recorded in a hospital study in Dhaka was in 1 to 2 year old [13] but in Kolkata, the peak age was in the 10 to 20 year group with no infections recorded in <1 year old [18]. There are three broad possibilities for this low incidence in very young children.

1. Lack of exposure to infection. Young children, especially breast fed babies, may not come in contact with infected water, food or environmental contamination and their parents may take extra precautions to prevent infection.
2. Innate or induced immunity including maternal immunity. Decay of maternal immunity would be consistent with the age distribution of typhoid seen in Dhaka but not Kolkata.
3. A normal susceptibility to infection, but symptoms so mild that they are not readily detected as has been hypothesized from a study in Chile [19]. However in other studies, in infants, symptoms have been severe [20] and case fatality rates high in younger infants [21].

This model provides ways of modeling each of these hypotheses by specifying.

- a refractory period without age specific exposure
- by specifying age specific exposure
- by specifying age specific morbidity.

In practice, a) and b) cannot be distinguished using age specific incidence (data not shown) and for simplicity in this paper only modeling outcomes using a refractory period are presented. Since adequate fits to the data are obtained with a), and c) would require a complex theory to explain differences in age specific morbidity in different communities, the most parsimonious approach has been followed by assuming c) is not generally operative. Thus in the modeling presented in this paper, over time a neonate becomes more likely to become infected (e.g. by increased exposure or loss of maternal immunity) then remains susceptible until an infection induces either clinical or sterile immunity.

Following a clinical infection, sterile immunity, which does not allow any subsequent infection, is induced with probability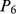. If the individual does not gain sterile immunity then the person may gain clinical immunity, which allows sub-clinical infections with probability 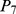. Similarly, following a subclinical infection in a non-immune person, sterile immunity is induced with probability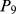 and clinical immunity, 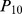. Exposure without becoming infected does not change immune status (i.e. exposure has no boosting effect on the immune status who has sterile immunity).

Infection induced sterile immunity always decays to clinical immunity (although this can be of zero duration) before it in turn decays to no immunity. A sub-clinical infection occurring while a person has naturally induced clinical immunity may boost immunity to the sterile category with probability 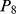 or increase the duration of clinical immunity by a duration sampled from user defined distribution, which may differ from the initial distribution of duration.

Although in this publication we do not present data on vaccination, for completeness of the description of the model, the immune transitions induced by vaccination, and subsequent loss of immunity are shown in Fig. 2B. For vaccination, only the final vaccination in the series is explicitly simulated if more than one injection is used so incomplete courses of vaccination have no effect. All simulated immunity starts in the time-step following infection/vaccination.

As for infection induced immunity, vaccines can induce either sterile immunity or clinical immunity. Sterile immunity decays to clinical immunity and then to no immunity. Again, similar to infection induced immunity, vaccination of a person previously vaccinated may boost immunity (from clinical to sterile immunity) and will extend the duration of immunity if there is pre-existing vaccine specific immunity, by a duration sampled from a different distribution to the original one. Vaccine induced immunity can be boosted in this way even in hosts with vaccine induced sterile immunity. In this model there is no direct interaction between clinical and vaccine induced immunity. For instance vaccination of a person who is already clinically immune from natural infections is just as likely to result in vaccine induced sterile immunity as vaccinating someone who is naive. Importantly, people who have vaccine induced clinical immunity, may be sub-clinically infected and thus acquire infection induced immunity.

### Case Management and Outcomes

A simulated acute clinical infection results one of four possible case management regimens, indexed by variable *d*. These are:

- untreated (*d* = 0);
- inadequate community treatment (*d* = 1);
- adequate community treatment (*d* = 2);
- treatment by a professional health care clinic or hospital (*d* = 3).

Each of these is associated with a different probability of dying from typhoid 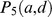 which may be age dependent. These simulated typhoid deaths are additional to those generated by the initial demographic model.

In addition to effects on survival, the different case management regimens modify infectiousness, sampled from distribution 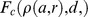 (Table 2). Relative to the distribution of 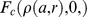 the infectiousness of adequately treated community cases sampled from 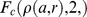 is generally reduced. Inadequate treatment, assumed to be using available antibiotics from informal sources, leads to substantial infectiousness (sampled from 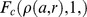. A hospital treatment is assumed to involve treatment with 2^nd^ and 3^rd^ line drugs if necessary. Patients become “quarantined” on admission so infectiousness, sampled from distribution 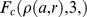 is generally lower than in the other case management categories, and subjects are unlikely to become temporary chronic carriers, so infectiousness it is limited to pre-admission time. All these infectiousness values can be age-specific but only age independent situations are modeled in this paper.

### Interventions

At any stage in the progression of the typhoid infection in the community (e.g. before any infection, during an epidemic, after achieving “stable” endemicity) an intervention can be introduced (e.g., vaccination, environmental controls, changed case detection and treatment). During these times, the population module continues to “grow” the population. The impact of an intervention program is assessed by computing the difference from a comparator population without the intervention.

A variety of different vaccination strategies can be modeled. These are any combination of:

1. Vaccination at a specific age (e.g. part of an EPI campaign or at school entry).
2. A mass vaccination campaign of all ages or of a specified age range.
3. Subsequent mass vaccination campaigns that can cover a the same or different age range as #2.
4. Booster vaccinations at a defined time intervals.

Coverage for initial vaccination and for boosters can be specified separately. Coverage in booster campaigns can vary from boosters for only those previously vaccinated to random coverage that includes those previously vaccinate and those unvaccinated.

Environmental interventions could include community based measures such as improved water supply or changes in individual behavior such as hand washing. Such measures are assumed to impact infectiousness, via the factor 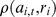 or the exposure of individuals in the distinct risk groups quantified by 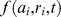. Temporal variation (in particular seasonal variation) in exposure is captured by 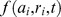.

### Reproduction Number from an Untreated Clinical Infection *R_c_*

In many transmission models, the critical transmission parameter is the Basic Reproduction number, R_0_. In this model, each of the infection states can have a different infectiousness, making R_0_ dependent on the type of infectious host. To encompass different types of infectious people, *R_c_* is defined as the number of new infections (subclinical and clinical) that would result in a completely naive population from a single acute untreated clinical patient of average age infected with a strain 1 *Salmonella* Typhi where both the infected and susceptible individuals are in a homogeneously mixed population. This reproduction number corresponds to the average infectiousness of acute clinical cases, 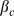, and relates to average levels of exposure and susceptibility (in the case of temporally invariant environments) via:
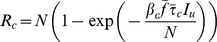
where 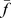 is the mean over the whole population of 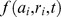, the scale factor measuring contamination of the environment, and 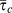 is the mean duration of infectiousness of the clinical cases. Infectiousness of other types of infection is expressed as a ratio of infectiousness per unit time to the reference infectiousness per unit time. In practice in this model, *R_c_* can be modulated in several ways:

1. This model allows two strains of Typhi to be simultaneously present. These can differ in infectiousness and clinical outcomes (e.g. as a result of different drug resistance). Both are tracked independently. The number of people infectious, their relatively infectivity and *R_c_* are then used to calculate the force of infection for the two types of Typhi present in the community in any month.
2. For each class of infection, the infectiousness can be specified as age dependent, if required.
3. For each class of infection, the exposure to infection can be age dependent, if required
4. The population does not have to be homogeneous with respect to exposure or infectiousness. A minimum of one homogenously mixed population class is required but the population can be divided in up to 10 classes each with their own exposure to and own contribution to the force of infection.
5. *R_c_* can vary with season.

This model assumes that there is a single common source of contamination accessible to all members of the community (with potentially different probabilities of contact) and that persistence of Typhi in the environment is short compared to the period of infection in individual. The model does not distinguish between modes of transmission (e.g. water born or direct contact).

The modeling presented in this paper uses a single strain of Typhi, and with the exception of an infant refractory/non-exposure period, a single homogeneous mixed population and unless specified, no variation of *R_c_* with season.

### Model Implementation

The model is implemented in Delphi 2005 (Borland Software Corporation, www.borland.com) and compiled to run on Windows and Linux based machines. Details of the input data requirements etc., in the File S2. Complied program and detailed instructions on running the program are available from the authors.

In this publication, the aim is to use the model to examine basic questions of typhoid biology and infection induced human immunity. Therefore only the simplest situation is modeled: one strain of Typhi; other than the neonatal “resistance to infection”, no age dependence of exposure or infectivity; a single class of population, no interventions. Unless otherwise specified, there is no seasonal variation in *R_c_*.

For the simulations of endemic typhoid reported here, the input parameters were assigned values by reference to the literature cited in the Table S1 in File S1. The model outputs were evaluated for.

- The incidence of typhoid reported from multiple sites (Table S2 in File S1).
- The average age of typhoid cases, and the age distribution of typhoid as reported by Saha et al for 2074 successive cases of typhoid collected from Dhaka from 1998 to 2004 (unpublished). The distribution is similar to an earlier set of 391 published cases [13].

For the incidence and age distribution and especially the former, the case definition of typhoid and the surveillance method impacts on the estimates. For most of the studies reported in File S2, the case definition was illness and a positive bacterial culture. There are a few reports from the same population where different case definitions were used. Siddique et al [14] measured an incidence of 170 per 100,000 per year in children <16 in Pakistan when using symptoms+blood culture and 700 per 100,000 per year when using symptoms+serology. Lin et al [22] compared incidence of cases of all ages with positive blood cultures (198 per 100,000 per year) in Vietnam and those diagnosed on the basis of symptoms (2323 per 100,000 per year).

Over a range of sites listed in Supplementary Table S3 in File S1, the highest incidence measured by culture positive cases over most of the population was 980 per 100,000 for people <40 in Kalkaji, New Delhi, India, an area with many migrants [20]. In areas where there was no reports of migrants, the highest culture positive incidence rates (per 100,000 per year) were 495: Ward 29 & 30 Kolkata [23], India; 405: Hijrat Colony, Sultanabad & Bilal Colony, Karachi, Pakistan [24]; and 370: Narkeldanga, Kolkata, India [18]. Since blood cultures are likely to underestimate typhoid cases, for a highly endemic area the incidence of clinical typhoid is at least 400 per 100,000 per year. A diagnosis on the basis of symptoms, or symptoms with serology may give an upper limit of the estimate of typhoid cases, the upper limit is likely to be about 2000 cases per 100,000 per year.

The case definition impacts on the parameterization of the model: the more stringent case definition requires a higher proportion of the infections to give rise to “subclinical” infections (i.e. those not recognized as Typhoid) and conversely the less stringent definitions of any symptoms requires a higher proportion of the infections to be recognized as cases. If it is assumed that every typhoid infection in a naïve person gives rise to an observable case as judged by symptoms (and that such a diagnosis has a high specificity), then the maximum proportion of naïve people with clinical cases as judged by blood culture could not be higher than 170/700 = 0.24 in the Siddique et al study or 0.09 in the Lin et al study and maybe lower if the sensitivity of detection of typhoid based on symptoms is substantially less than 100%. In the modeling presented below, we use a probability of detecting an infection of 0.1 assuming a case detection based on culture and therefore use an incidence range of 300 to 600 cases per 100,000 per year as a constraint on the feasible parameter space.

The reported average age of cases varies from study to study (Table S2 in File S1). In determining a feasible parameter space we first use the average age of the Dhaka data set (76 months), then the age distribution of typhoid from this data set to determine a feasible parameter space for Dhaka, then extend this to encompass a data set from Kolkata [18].

Maximum likelihood estimation (MLE) was used to determine the best fit for *R_c_*, the Infant refractory/non-exposure period and its standard deviation for the Dhaka and Kolkata data and for the Kolkata data the phase and amplitude of seasonal variation in *R_c_* assuming that the cases observed in each age bracket or each month followed a Poisson distribution. Confidence intervals for the estimated parameters were derived from the second derivative of a second order polynomial fit to the log likelihood values as a function of the parameter being estimated.

## Results

### Plausible Parameter Space for Stable Endemicity based on Dhaka Data Set

A series of simulations were performed to systematically vary *R_c_*, the duration of “infant resistance/non-exposure”, the duration of clinical and sterile immunity and the probability of acquiring clinical and sterile immunity for clinical and subclinical infections.

For the results reported here for modelling stable endemic typhoid in Dhaka and in Kolkata the force of infection was incremented by the amount equivalent to that resulting from a single acute infection each year for 40 years then the population followed for a further 40 years without further introduction.

The outcome of the simulations were then examined for their ability to predict.

1. The average age of infection observed in the Dhaka data set,
2. The distribution of the age of infection in Dhaka.
3. The incidence observed in the studies reference in “Model calibration”.

**Figure 3:**
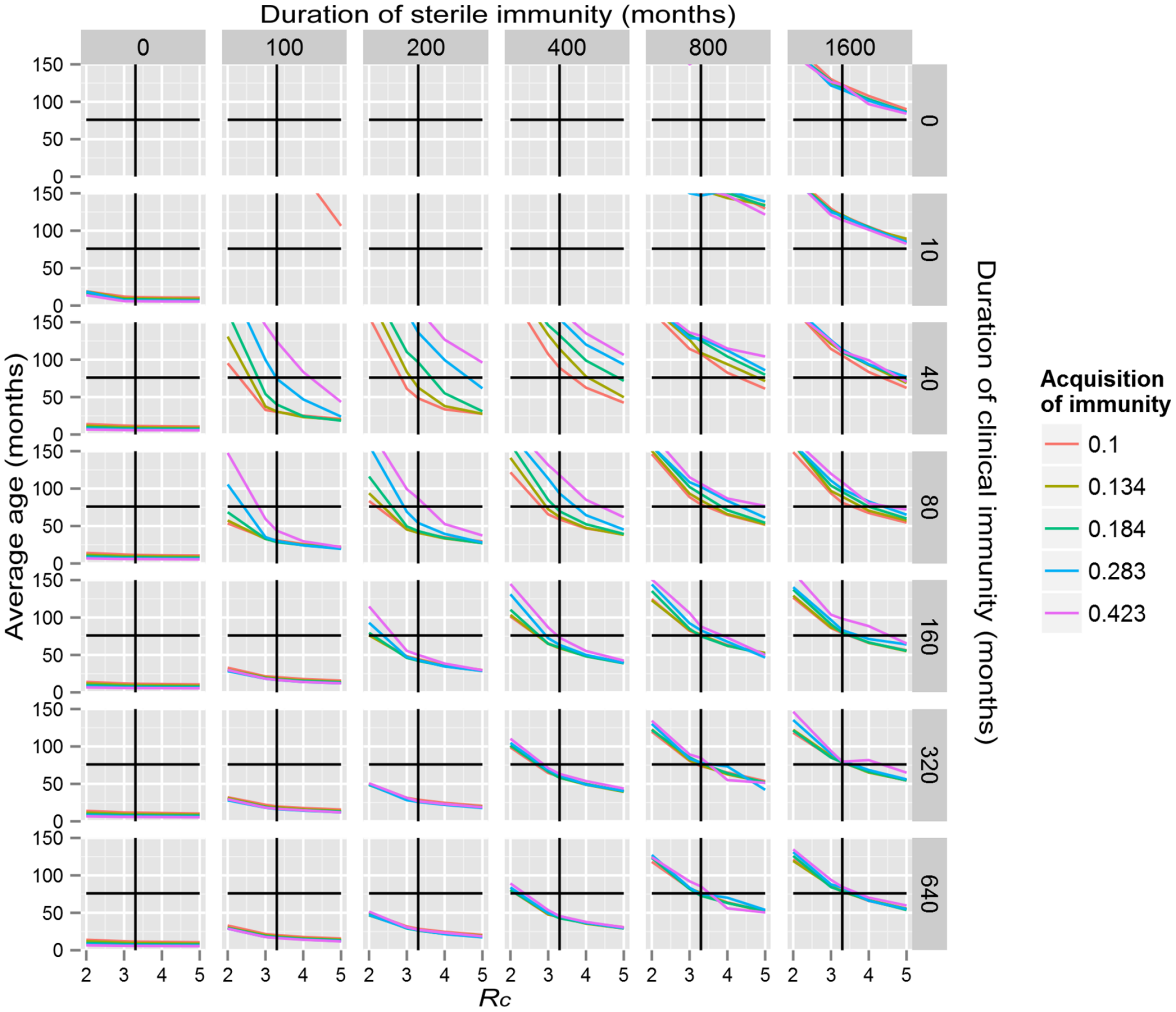
Sensitivity analysis: average age. Change in average age as a function of probability of acquiring immunity, duration of sterile and clinical immunity and *R_c_*. Within each graph the probability of acquiring immunity of 0.1, 0.134, 0.184, 0.283, 0.422 for the purple, blue, green, orange and red plots, respectively with an equal probability of acquiring sterile or clinical immunity (conditional on not acquiring sterile immunity) from either a subclinical or clinical infection. These probabilities correspond to an average of 10, 5, 4, 3, 2 and 1.5 infections required to acquire immunity, respectively. Graphs in each column used the same duration of sterile immunity; graphs in each row used the same duration of clinical immunity. Horizontal black line is an average age of 76 months and black vertical line an *R_c_* of 3.3, the initial estimate of these values for Dhaka.

One set of slices through the resulting hypercube is shown in Fig. 3 to examine the average clinical age as a function of *R_c_*, duration of clinical and sterile immunity and assumptions about the probability of acquisition of clinical and sterile immunity. As shown in this diagram, all combinations tested of *R_c_*, with an assumption about acquisition of immunity with a very short duration of clinical immunity (0 months) gave a predicted average age of infection greater than the observed average age of 75 months. Similarly, all combination tested with long durations of clinical immunity but a relatively short duration of sterile immunity (200 months or less) gave an average age of less than the observed 75 months. For simulations with a long duration of sterile immunity (800 months or longer) there was relatively little impact on assumptions about the number of clinical or sub-clinical episodes prior to gaining immunity on the average age of infection. In all these simulations, the average age of infection is highly dependent on *R_c_*, as has been reported by Crump et al [23].

**Figure 4:**
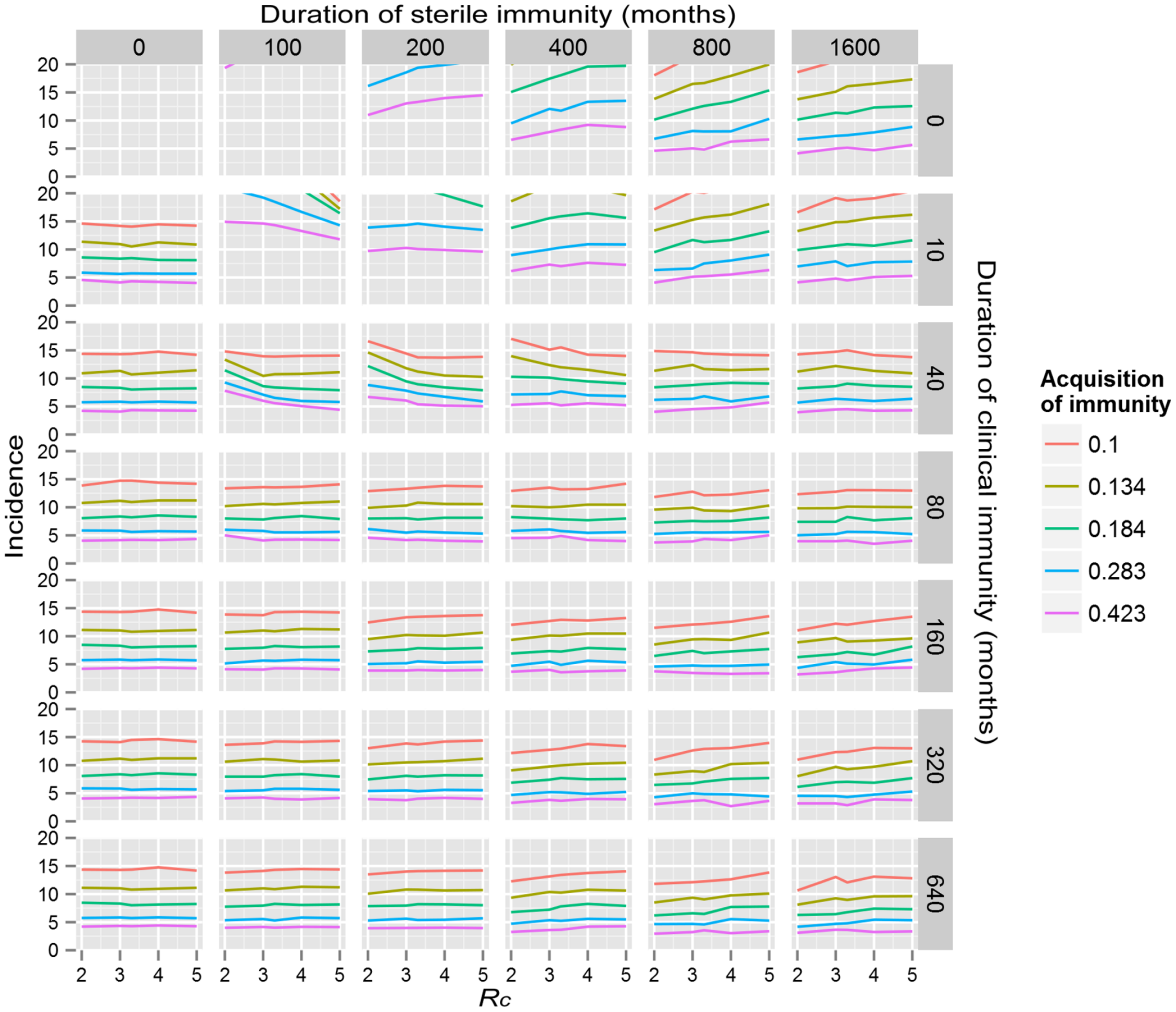
Sensitivity analysis: Incidence. Change in incidence as a function of probability of acquiring immunity, duration of sterile and clinical immunity and *R_c_.* Incidence is clinical cases per month per 1000 population. Parameters used for and the organizations of the graphs are the same as for Fig. 3.

By contrast (Fig. 4), *R_c_* had little impact on the estimation of the incidence of clinical typhoid and provided there was at least some duration clinical immunity (40 months or greater), the duration of immunity also had little impact on the estimates of incidence. However, assumptions on the number of clinical or subclinical episodes required to induce immunity had a major impact. On the basis of these data, two of the sets of assumptions about acquisition of immunity seem implausible. The assumption that a low proportion (<33% ) of clinical or sub clinical episodes of typhoid give rise to either clinical or sterile immunity leads to a predicted incidence rate substantially greater than that observed. Similarly, the assumption that every episode gives rise to either sterile or clinical immunity leads to incidence rates for feasible durations of sterile or clinical immunity that are too low. This is consistent with biological data showing that most people in clinical trials or in recurrent epidemics can be infected more than once (see Table S1 in File S1for details).

**Figure 5:**
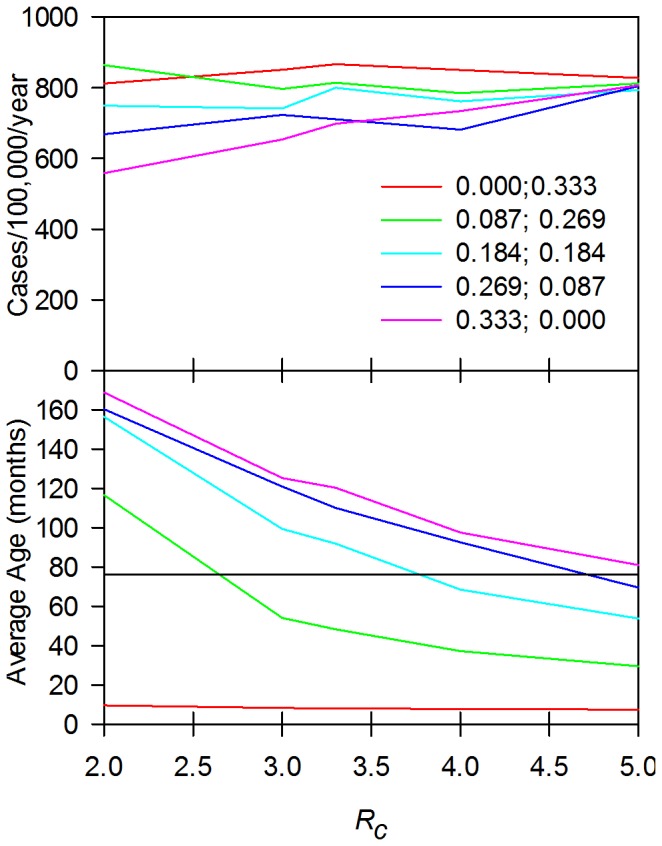
Impact of type of immunity induced by infection as a function of *R_c_.* Impact on incidence (upper panel) and average age (lower) panel. The standard parameters for Dhaka were used assuming an average of three infections was required to induce immunity. For each line, the first number is the probability of inducing sterile immunity and the second the probability of inducing clinical immunity, conditional on not inducing sterile immunity. In these simulations, the probability of inducing immunity by a subclinical infection or clinical infection was assumed to be similar. Thus 0.333, 0.000 is a simulation that only induces sterile immunity; 0.000, 0.333 only induces clinical immunity. The horizontal black line in the lower panel is the average age of infection (76 months) observed in Dhaka.

Further refinement of these data by examining the predicted incidence and average age for simulations where a range of combinations of 3 subclinical or clinical infections, were required on average to give immunity were examined (Fig. 5) with medium length clinical immunity (160 months) and long lived sterile immunity (800 months). In this case, all simulations gave similar incidence regardless of *R_c_*, but the average age of infection was now dependent not only on *R_c_* but also on the balance between the extremes of infections only giving rise to clinical or only to sterile immunity. Consistent with the results shown in Fig 3, infections that only gave rise to clinical immunity, or a low probability of sterile immunity gave average ages of infection that were unrealistically low. Infections that only gave sterile immunity gave average infection ages higher than expected, while infections that had a similar probability of giving rise to either sterile or clinical immunity gave average ages that included the observed average age in Dhaka.

**Figure 6:**
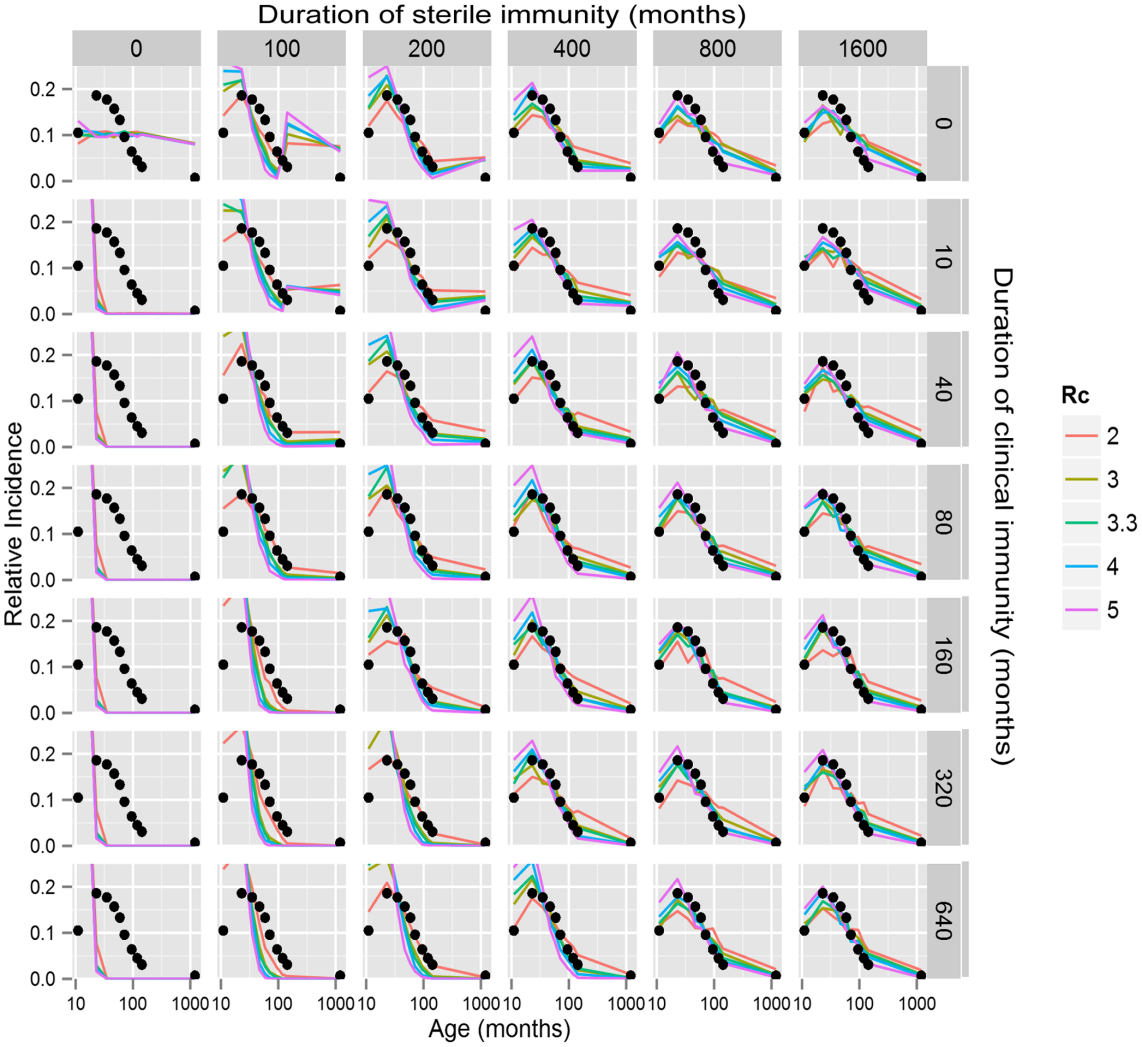
Sensitivity analysis: Age distribution. Age distribution was calculated as a function of duration of sterile and clinical immunity and *R_c_*. Probabilities of acquiring immunity were 0.184 for sterile or clinical immunity and the duration of infant refractoriness/non-exposure was 8±6 months. Within each graph the observed distribution (black diamonds) and predicted distribution is plotted vs log age (years). *R_c_* was 2, 3, 3.3, 4 and 5 for the purple, blue, green, orange and red plots, respectively.

Using average age and incidence only uses part of the data available from Dhaka. More information is available in the age distribution of typhoid. In particular the whole age distribution allows estimates of the “infant refractory/non-exposure period” as well as better estimates of the likely *R_c_*. Fig. 6 illustrates a set of simulations in which the age specific relative incidence is compared to the observed relative incidence (since the denominator is not known for the Dhaka data, only relative incidence is available). These simulations rely on an estimated duration of the infant refractory/non exposure period of 8±6 months obtained from earlier fitting (data not shown). In these graphs, the black diamonds are the observed data and the incidence is plotted as a function of age for different assumptions about *R_c_*, and duration of sterile and clinical immunity.

*Conclusions from sensitivity analysis for fitting parameters to Dhaka data.* Once induced, sterile immunity is long lived (400 months or longer). Clinical immunity is likely to be 40 months or greater. People (usually children) will have 2 to 3 typhoid infections before developing immunity. From this analysis of the parameter space, we chose the standard set of immunological parameters in Table 4.

**Table 4:** Standard immunological parameters.

Standard Parameter	value
Probability of acquiring sterile immunity from a clinical episode	0.184
Probability of acquiring clinical immunity from a clinical episode conditional on not acquiring sterile immunity	0.184
Probability of acquiring sterile immunity from a sub-clinical episode	0.184
Probability of acquiring clinical immunity from a sub-clinical episode conditional on not acquiring sterile immunity	0.184
Probability of acquiring sterile immunity from a sub-clinical episode in a person with clinical immunity	0.184
Duration of clinical immunity (months)	160
Duration of sterile immunity (months)	800

Since these parameters primarily relate to the human immune response to infection with typhoid and not the environmental parameters, these should be applicable to most populations. However, the lack of detailed field data limits the precision with which this parameter space can be defined. Note that with the limited data it is possible to neither define the uncertainty in these parameters nor estimate for example, the deviation in the duration of immunity in the populations.

**Figure 7:**
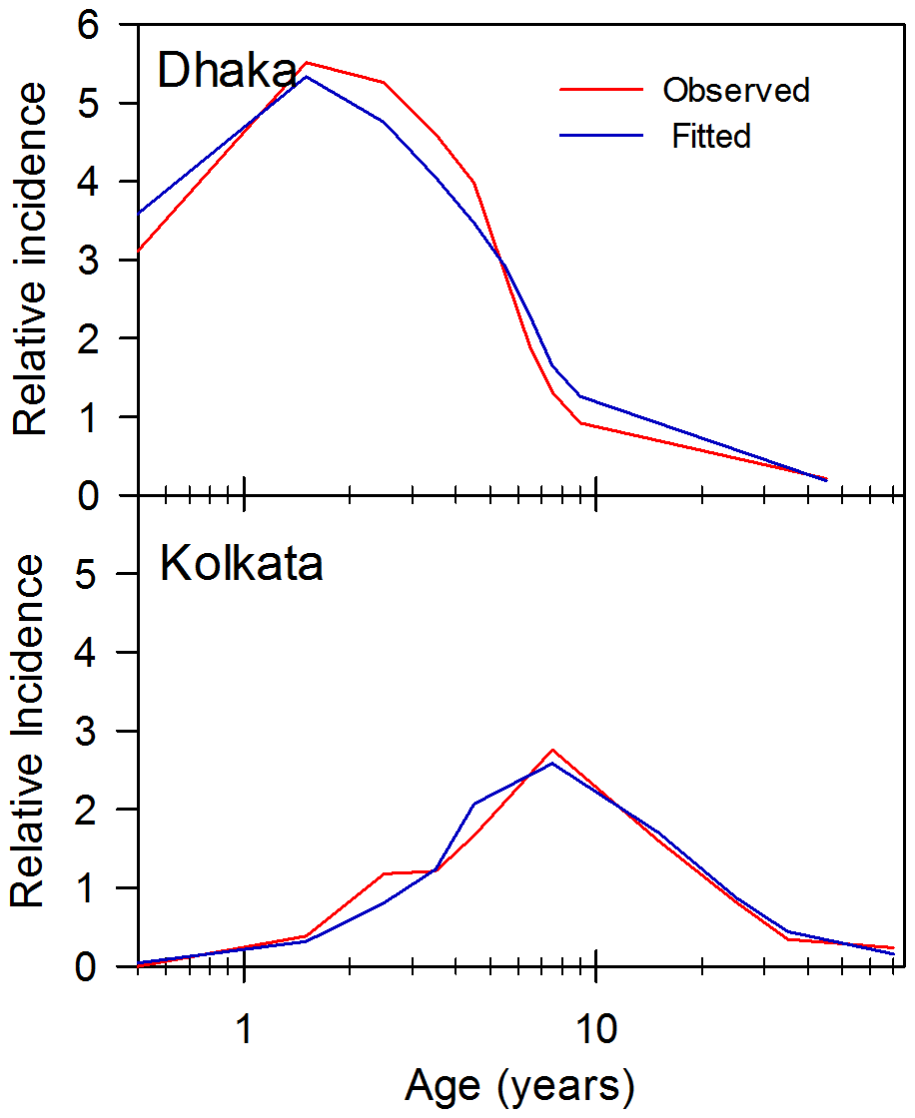
Comparison of the simulated and observed age distribution of typhoid cases in Dhakaand Kolkata. The simulated distributions were calculated using the estimates obtained by maximum likelihood estimations for *R_c_*, infant protection/non-exposure period and its standard deviation. *R_c_* for Dhaka (upper panel) was 3.4 and 2.1 for Kolkata (lower panel). Infant protection/non-exposure period and their standard deviations were 8±6 months and 77±37 months for Dhaka and Kolkata, respectively. We assume that other parmeters (e.g. probability of acquiring immunity following an infection) are common to both sites and are specified in the text. The age-specific incidence is plotted relative to the overall incidence in the population of 1.

Using the parameters in Table 4, the best MLE estimates for Rc, the infant refractor/non-exposed period were re-evaluated and remained similar to the earlier estimates used for the sensitivity analyses in Figs. 3 and 4 with an estimated infant refectory/non exposed period of 8.6 (95% CI ±1.2) months with a standard deviation of the infant refractory/non-exposure period of 6.4 (95% CI ±2.4) months. With these parameters the best estimate of *R_c_* is 3.4 (95% CI ±0.14). Using these estimates, the predicted age distribution compared to the measured distribution is shown in the upper panel of Fig. 7.

### Extension to other Situations

#### Kolkata

**Figure 8:**
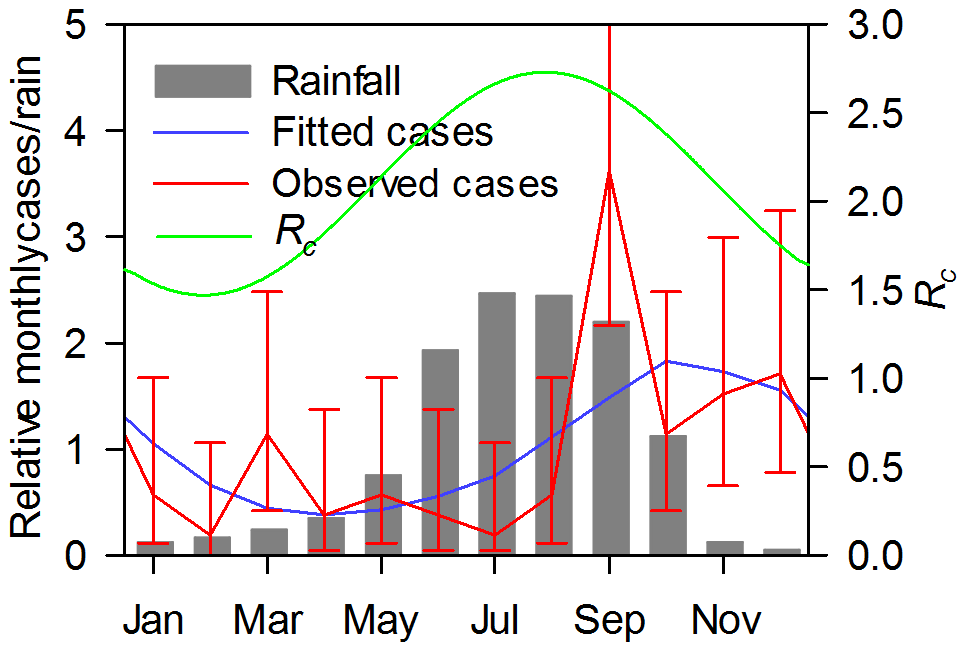
Comparison of the simulated with observed seasonality of Typhoid in Kolkata. The simulations approximate the seasonality of *R_c_* with a sine function (green curve). The best fit (blue plot) to the observed data (red plot) was obtained with an average *R_c_* of 2.1 and a peak *R_c_* of 2.73 (95% CI ±0.30) on 12^th^ August (95% CI: ±24 days). Average monthly rainfall for Kolkata is shown with the grey bars. Cases are plotted relative to the observed monthly average of 6.25 and rainfall relative to the average monthly rainfall of 134.5 mm (www.imdkolkata.gov.in).

Age specific incidence was obtained from Kolkata [18] prior to the instigation of a large cluster randomized trial [25]. The 94 cases were collected over a period of one year and it was apparent that there was a marked seasonality to the observed incidence of cases. In this case, the model was used to estimate infant refractory/non-exposure period, standard deviation of the infant refractory/non-exposed period and *R_c_* (76.5±18.0 months, 37.1±7.5 months and 2.1±0.36, respectively). Using these estimates, the predicted age distribution compared to the measured distribution is shown in the lower panel of Fig. 7. The Kolkata cases showed a marked seasonal variation and these data were fitted to a seasonal model assuming a sine variation. In the published data, the month in which 63 of the cases occurred are specified. Multiple underlying models could be chosen but the sine function was chosen since it is the most parsimonious, requiring only two parameters : an amplitude and a phase parameter. As shown in Fig. 8 the sine function gave a reasonable fit with peak *R_c_* of 30% (95% CI ±14%) higher than the mean *R_c_* of 2.1 and occurring on August 12 (95% CI ±28 days). The fitted model is within the 95% confidence intervals for the estimated true cases for each month except for September (Fig. 8). Importantly, the peak *R_c_* occurred approximately 2.5 months before the peak in incidence and coincided with the period of peak rainfall. However, this model predicts a substantial year round component for *R_c_*, even though rainfall drops to low levels from November to February [26].

### Variation in R_c_ and the Probability of Developing a Clinical Infection

**Figure 9:**
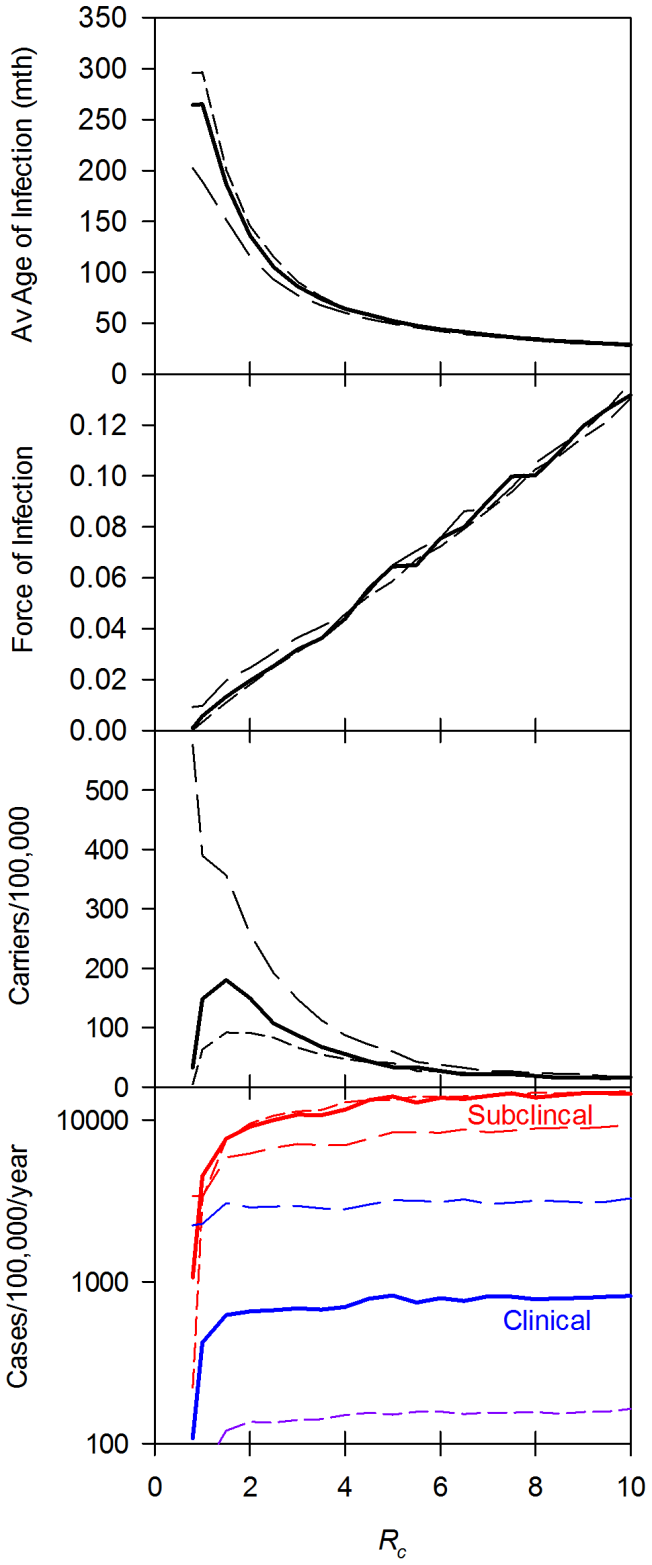
Effect of varying *R_c_* and the proportion of infections giving clinical cases. The simulations systematically *R_c_* for probabilities of 0.02 (short dash), 0.1 (standard conditions – thick continuous line) and 0.5 (long dash) of an infection in a non-immune person with the standard Dhaka parameter set for other parameters, showing the average age of infection, the force of infection (infectious dose per person per month), the number of chronic carriers per 100,000 and the number of subclinical and clinical cases per 100,000 per year.

The average age, the force of infection during the last year of the simulation, the point prevalence of the chronic carrier states and the incidence of both sub-clinical and clinical disease at the end of the simulation, were calculated for a series of different *R_c_* values and for the *P_1_* (Fig. 1), the probability of an infection in a naïve person becoming a clinical case using values of the other parameters used for fitting the Dhaka data (Fig. 9). The average age of infection decreased with increasing *R_c_*. At the lowest *R_c_* that gave sustained transmission, the average age of infection was approximately the average age of the population, consistent with a lack of immunity in the population under very low transmission conditions. Varying *P_1_* over a 25 fold range from 2% to 50% had little impact: At *P_1_* of 0.5 there was a small decrease in the average age at low *R_c_*. This reflected a decrease in the average age of the population due to the relatively high death rate specifically due to typhoid compared to a *P_1_* of 0.02 and 0.1.

The Force of Infection was linearly related to *R_c_* in cases where an endemic stable state was achieved, with a critical lower value of R*_c_ = 0.69 at *P_1_* of 0.1. Further simulations (not shown) demonstrated that the threshold was dependent on the assumptions about the probabilities of carriers. In simulations with no carriers, further simulations (not shown) demonstrated that the threshold proved insensitive to assumptions about the refractory period but was dependent on the assumptions about the probabilities of carriers. In the absence of carriers, R*_c_ = 1.0. R*_c_ was not dependent on assumptions about the period of the infant refractory/lack of exposure period. This is unsurprising since the latter applies only to the youngest age groups, and at low levels of transmission the disease occurs sporadically in all ages, and so transmission does not depend on susceptibility of infants. The relationship between Force of Infection and *R_c_* did not show a major change over the range of *P_1_* considered. However, as discussed below, the number of chronic carriers was sensitive to *P_1_* and as a result, although there was a small shift at low Rc reflecting the increased transmission from carriers with R*_c_ = 0.76 at *P_1_* of 0.02 and R*_c_ = 0.29 at *P_1_* of 0.5.

For *P_1_* of 0.1, the prevalence of chronic carriers peaked at around *R_c_* = 1.5, with higher transmission levels associated with lower carrier rates. Relatively few carriers were observed in simulations of highly endemic areas. Since this model assumes that the chronic carrier state can only arise from a clinical infection, it is not surprising that the number of carriers in the community is highly dependent on *P_1_*. Incidence of both sub-clinical and clinical disease, initially increased with *R_c_* but as seen in the sensitivity analysis, plateaued and again, as expected, the incidence of clinical cases is nearly proportional to *P_1_* The incidence of sub-clinical infections, while showing some decrease at a *P_1_* of 0.5, is much less dependent on P1 since many of these subclinical infections occur in people with clinical immunity.

**Figure 10:**
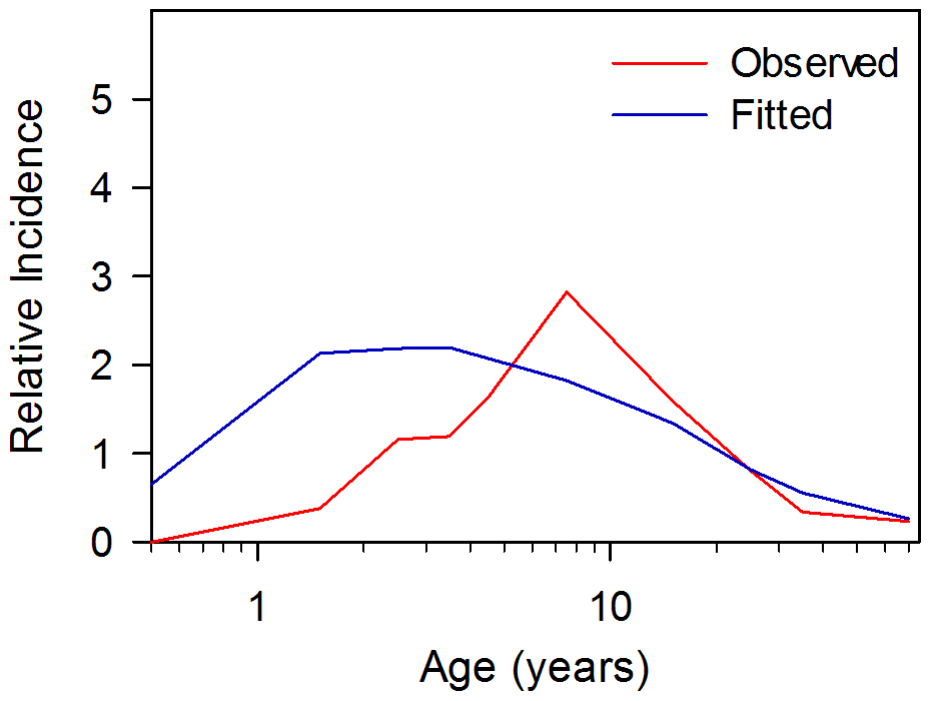
Inability of variation in *R_c_* to explain differences in age distribution in different populations. Simulations based on the Dhaka data set, including an infant refractory/lack of exposure period of 8±6 months with *R_c_* chosen to give the observed average age of infection in Kolkata of 15 years. Note that this simulation compared to the simulation shown in Fig 7, over-predicts the number of young children with symptomatic typhoid.

Although the average age of cases depends on *R_c_*, *R_c_* is not the only determinant of this age distribution. Using the Dhaka set of parameters, an *R_c_* of 1.9 gives the average age cases of 15 years observed in the Kolkata data [18]. However as shown in Fig. 10, the distribution observed in Kolkata is poorly fitted using an *R_c_* of 1.9 and the Dhaka estimates of the refractory/non-exposure period. As this example illustrates at low values of *R_c_*, and particularly as *R_c_* approaches R_c_*, the incidence ceases to become a function of age.

### The Role of the Carrier States

To investigate the role of the carrier states, the simulations based on Dhaka were re-run, using fixed immunological parameters, but this time varying the probability of becoming a temporary or chronic carrier, the duration of temporary carriage, *R_c_* and the population size and a similar analysis conducted as describe above (i.e. impact of varying parameters was examined on average age, overall incidence and then for selected combinations.

**Figure 11:**
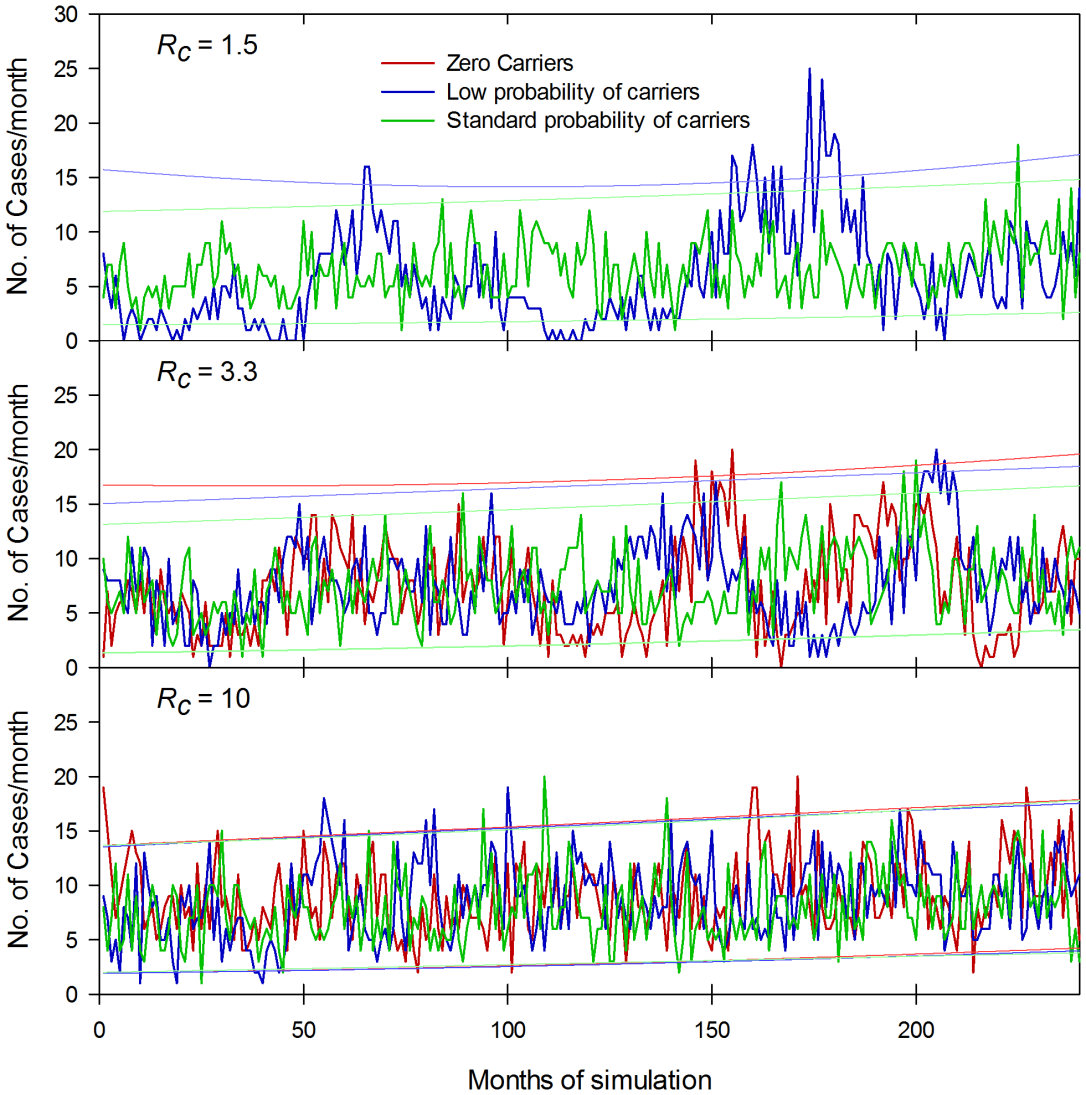
Impact of carriers and *R_c_* on the stability of time series of typhoid cases in an endemic community: individual simulations. Individual simulations are shown of the number of cases per month for a 240 month (20 year) simulation period. Simulations used the basic Dhaka parameters but varied *R_c_* (1.5: top panel, 3.3: middle panel and 10 lower panel) assuming a zero probability of becoming a carrier (red plot), 12.5% of standard probability of becoming a carrier (blue plot) and the standard probability (green plot). At low transmission (*R_c_* 1.5) in the absence of carriers, the infection becomes epidemic and does not persist, hence only low and standard probability traces are present in the top panel. The 95% confidence limits on the maximum and minimum cases per month are shown by the thin red, blue or green lines for the corresponding probabilities of becoming a carrier derived from 200 simulations. The lower 95% confidence is zero for zero probability of carriage at an *R_c_* of 3.3 and zero for 12.5% probability for and *R_c_* of both 1.5 and 3.3 and are not shown on these graphs. In each simulation the starting population was approximately 10,000 growing to 16,000 after 20 years.

**Figure 12:**
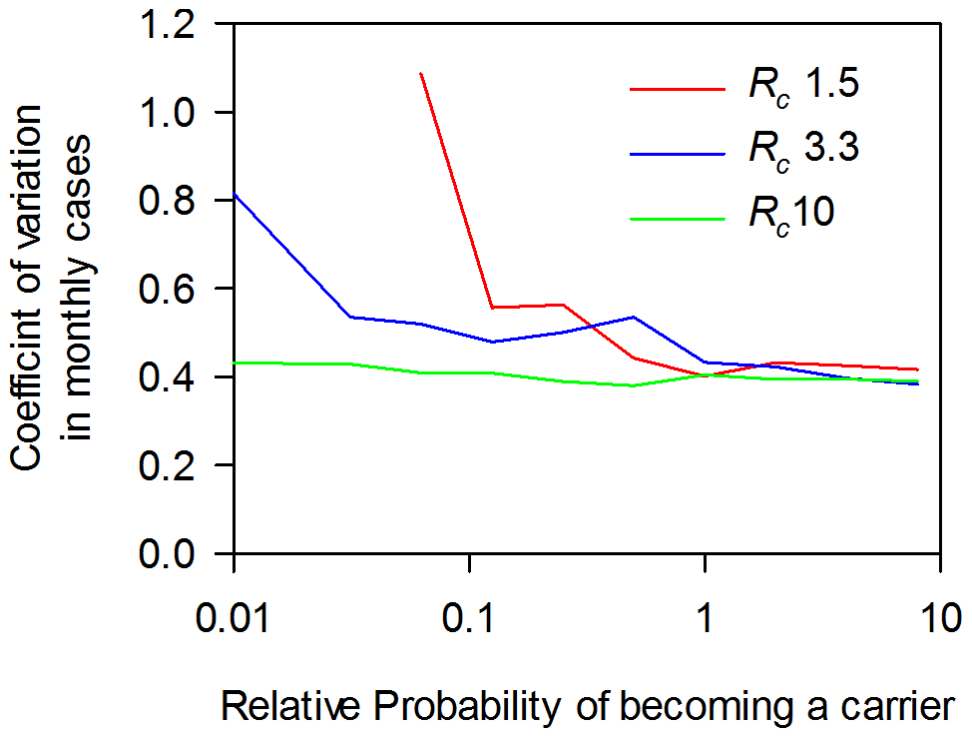
Impact of carriers and *R_c_* on the stability of time series of typhoid cases in an endemic community: variation in the number of monthly cases. Analysis of simulations using similar parameters to Fig. 11, showing the coefficient of variation in the number of monthly cases and using a more detailed range of probabilities of becoming a carrier. As for Fig. 11, endemic typhoid was not possible at low values of *R_c_* and at low probabilities of becoming a carrier.

When simulating the Dhaka data, we can find no significant effect when the probability of becoming a temporary carrier is varied from zero to one, consistent with the small contribution of these carriers to the overall reservoir of infection. This does not mean that they make no contribution in Dhaka, but the impact on the force of infection cannot be distinguished from minor changes in *R_c_*. On the other hand, the role of chronic carriers was critical for stability of the endemic state: in the absence of chronic carriers especially for low values of *R_c_*, stochastic fluctuations in the number of people infected frequently led to elimination of the infection in the community. At very low probabilities of an adult becoming a chronic carrier, the models predicted major fluctuations in the incidence of clinical typhoid and this depends on *R_c_*. Fig. 11 shows monthly number of clinical cases for individual simulations over a period of 20 years, starting with a population of approximately 10,000 growing to a population of 16,000 at the end of the simulation over a range of probabilities of a naïve adult becoming a carrier following a single infection. Individual simulations are shown for *R_c_* of 1.5, 3.3 and 10 for zero probability of becoming a chronic carrier (*R_c_* 3.3 and 10 only) and for 1/8 of the standard probabilities. For *R_c_* of 1.5, the infection failed to persist in 0, 21, 27, 44 and 50 attempts out of 50 runs with probabilities of 0, 1/32, 1/16, 1/8, 1/4, respectively, of the “standard” probability for developing chronic carriage used in the simulations of 0.04 and 0.0128 per case for female and male adults (see Table S1 in File S1, for derivation of the standard probability). One measure of the fluctuations is the coefficient of variation in the monthly incidence of clinical cases for infections that persisted in the community over the full 40 year simulation period (Fig. 12). At an *R_c_* of 10, as judged by the CV of the incidence, the presence or absence of chronic carriers had no effect on the stability of the typhoid. At an *R_c_* of 3.3, even low probabilities of becoming a carrier removed most of the fluctuations with time and reduced the CV. (e.g. the monthly incidence was relatively stable at the lowest probability tested of 1/32 of the “standard” estimate).

## Discussion

The agent based simulation model for Typhoid presented in this publication illustrated both the complexity of typhoid transmission and disease as currently understood, and also major gaps in our knowledge. In particular, the model is able to potentially accommodate the multiple classes of infection (incubating, subclinical, acute, temporary carriers/relapsing infections and long term chronic carriage), different levels of immunity (clinical and sterile immunity), migration of naïve or infected individual into the community and different levels of infectiousness for individuals and inhomogeneity in the community. Extensions of the model are designed to model different intervention strategies.

As for many models, the use of this model is limited by the available data against which it can be calibrated. However, it is able to closely simulate the age distribution of typhoid cases seen in different endemic settings (Dhaka and Kolkata). With the inclusion of the infant refractory/non-exposure period, the model gave a good fit to both the Kolkata and Dhaka data sets assuming uniform exposure of the population Thus with the available data, there is no justification for using more complicated versions of this model that allow structuring of the population into subpopulations nor for using different models that explicitly include preferential clustering such as has been used for investigating the impact of contacts on influenza transmission [27].

In studies beyond the scope of this paper, the structure of the model could allow modeling of communities stratified according to distance from a contaminated water supply, or by socio-economic status or by age where data suggested that transmission was primarily within a defined age group, e.g. school age children. For example, it would be interesting to see if this model can predict the spatial distribution observed in studies in Kathmandu where the incidence of Typhi and Paratyphi A varied according to environmental factors [28].

Since all transmission is assumed to take place via a common source of infection, one limitation of the current implementation of this model is that multiple discrete transmission cycles cannot take place within a single community (i.e. there cannot be a school cycle occurring largely independently from a contaminated water cycle).

The basic model addresses three important aspects of the biology and immunology of typhoid infections.

1. There is a marked difference in the number of cases of typhoid seen in infants and young children in Dhaka and Kolkata. The data from Dhaka show an average age of clinical cases at 76 months, a peak age in clinical incidence of approximately 24 months and 49% of cases in children <48 months. Similar young ages are seen in data from coastal areas near Karachi, Pakistan [15], from New Delhi, India [29] and from Nigeria [17]. This contrasts strongly with data from Kolkata where infections are rare in young children (7.4% clinical cases in children <48 months). Few infant infections were also seen in a dataset of 2762 cases in Kathmandu with only 7.0% of clinical cases occurring in children <48 months. Although it has been well documented that the average age and more specifically the age distribution of cases depends on the transmission rates [30], the modelling presented here suggests that these differences in the transmission rates between the Dhaka, and Kolkata cannot be due to differences in transmission as encompassed by *R_c_*. Other explanations, e.g. different exposure of the young children to typhoid, must be considered. This has major implications for control strategies, especially for vaccine deployment. Vaccination of children at age about 4 years in Kolkata would cover the 93% of the population currently at risk of typhoid. In Dhaka, vaccination of children at age 1 year would be required to achieve the same results.
2. These studies highlight our lack of knowledge about the acquisition and loss of infection induced immunity. The modelling suggests that multiple (e.g. approximately 3) infections are required to become functionally immune to typhoid and this is consistent with field data [2] and limited data from challenge experiments (and summarized in S1). There are no direct measurements of the duration of immunity from field data. However within the constraints of the assumptions of the model, the simulation studies show that both clinical and long lived sterile immunity is required in order to generate the incidence and age distributions of cases observed from endemic populations.
3. The modelling highlights the importance of chronic carriers to the stability of typhoid in endemic situations. Without carriers, typhoid infections resemble the pattern of infections seen by diseases such as measles: characterized by local epidemics and requiring a large effective population size for maintaining infection. However even with low probabilities of becoming a carrier, there is a marked stabilization of the endemicity. Paradoxically, the model predicts that the frequency of carriers will be inversely dependent on the transmission rates of typhoid with the highest frequency found in low endemic areas with a relatively high incidence in adults. This model prediction is consistent with the apparent high frequency of carriers observed in a relatively low endemic area of Chile [31], but the inability to find any carriers in a highly endemic area of Vietnam [32].

In addition to the insights from this model to the basic biology of typhoid infections, this model is intended as the framework for future studies on designing responses to control outbreaks of typhoid fever and for controls for reducing the impact of endemic typhoid. In particular, with the likely introduction of new generations of typhoid vaccines in the near future, this model may contribute to planning of both typhoid vaccine trials and the development of cost effective implementation strategies.

## Supporting Information

File S1 **Table S1. Ranges of Parameter values used in the model with literature review justifying choices.** Table S2. Typhoid incidence estimates from locations with endemic typhoid fever. • Table S3. Literature values for case fatality rates.
(PDF)

File S2 **Description of software, input and output files.**
(DOCX)
