# Supplementary material for: Stochastic Simulation of Endemic Salmonella enterica Serovar Typhi: The Importance of Long Lasting Immunity and the Carrier State
Source: PLoS One. 2013 Sep 10;8(9):e74097. doi: 10.1371/journal.pone.0074097 (PMC3769365; doi:10.1371/journal.pone.0074097)
Supplement: File S1 — Table S1. Ranges of Parameter values used in the model with literature review justifying choices. Table S2. Typhoid incidence estimates from locations with endemic typhoid fever. • Table S3. Literature values for case fatality rates. (PDF) [file pone.0074097.s001.pdf]

Table S1. Default Parameter values and justification

Symbol	Name	Default Value	Justification
P_1	Proportion of infections in a non-immune people that lead to documented disease	0.1	***Assumed from literature but also subjected to sensitivity analyses covering range 0.02 to 0.5***<br>In any one time step the probability a person is infected depends on λ, the force of infection, and their immune status, with individuals of the same age within a single subpopulation having the same probability of infection provided they are not resistant or have infection or vaccine induced sterile immunity. In people that have infection or vaccine induced clinical immunity, all of these newly infected people will develop a sub-clinical infection. In people with no immunity, of those who are infected, P_1 will develop a clinical infection, the remainder a sub-clinical infection.<br><br>Two field studies that compared a culture confirmed diagnosis with a diagnosis based on symptoms or serology suggested that a document clinical case definition based on a positive culture may only detect 24% [1] or 8.5% [2] in people with at least some symptoms, and presumably even lower proportions if completely asymptomatic infections are included.<br><br>In human challenge studies there was a clear relationship between the number of bacteria ingested and the probability of developing clinical infection. The default value has been chosen on the assumption that in the field most of the infections will result from relatively low inocula. This assumption can be partially justified on a comparison of dose, incubation time for clinical typhoid and % ill in volunteers (4.7, 7.4 and 9.3 days with 96%, 48% and 41% for inocula of 10^8, 10^7 and 10^5, respectively) [3] compared to the average incubation time of 13.2 and 18.5 days with 16.7 and 3.8% attack rates estimated from food and water borne epidemics [4].<br><br>Indirectly one can conclude that the proportion of people who had sub-clinical infections shows a bell shaped distribution – at very low doses, the low force of infection will result in few infections – either clinical or subclinical. At high doses, the force of infection is sufficiently high that almost all people will be infected, but as almost 100% will have clinical infections, the proportion with sub-clinical must be low. The model structure that makes subclinical infection conditional on not having a clinical infection mirrors this conclusion.<br><br>Although the incubation period depended on the size of the inoculum, the severity of the disease did not [3,5,6], a finding also found from analyses of naturally occurring outbreaks of disease [4,7].

P_2	Proportion of sub-clinically infected that become life-long carriers	5% of probability from a clinically infected case	***Assumed from literature values***<br>Although there are reports in the pre-antibiotic era of carriers with no record of a previous episode of typhoid fever, these were uncommon [8]. Probability assumes that the literature values of 20% of carriers with no prior history overestimates probability per infection since cases of prior typhoid may have been unrecorded or misdiagnosed and that in a clinically immune person there may be many episodes of sub-clincial infections.
P_3	Proportion of clinically infected patients that become life-long carriers	Age dependent table, distribution<br>Adult female 0.04<br>Adult male 0.0124<br>Average 0.026	***Derived from literature values based on data from Santiago[9];***<br>The probability of becoming a carrier depends on age and gender, with women about 3 times more likely than men [8]. It is highly associated with infection of the gall bladder [8] and one likely hypothesis is that Typhi forms biofilms on gall stones [9]. In the pre-antibiotic era, remove of the gall bladder was used to “cure” chronic carriers [10]. Based on the association with gall bladder infection, from autopsy data, Levine et al were able to calculate carriage rates in Santiago, Chile and estimated the frequency at 695 per 100,000 with carriage rates as expected, much higher in females than males [11]. However, gall bladder infections are not the only sites that can become chronically infected, removal of the gall bladder from chronic carriers did not always cure carriage [10] and chronic urinary secretors are also known [8] . Interestingly, vertically transmitted neonatal infections can also become chronic carriers [12], so although carriage is predominantly in the adult population, younger carriers are not impossible. Estimates of the probability that people with typhoid fever progress to becoming chronic carriers come from studies of typhoid cases. In a series of 163 male soldiers infected in a prisoner of war camp in New York, Garbat [13] estimated 2.4 to 4.2% became carriers and quotes data from contemporary sources estimating approximately 4% of cases become carriers.<br><br>In a series of 2181 cases from New York State from 1930 to 1934, 54 (2.5%) remained infected at least a year after their disease [14]. In a different analysis of data from New York State, Ames et al demonstrated a marked age dependency on the frequency with which people infected between 1930 and 1939 became carriers [15]. Overall they saw a similar frequency (2.9%) to the Stebbins et al study [14] in the 3130 patients in their series. However the proportion of cases becoming chronic carriers varied from 0.3 in the <10 and 10-19 age groups, through 2.1% in the 20 to 29 and 4.4 in the 30 to 39 age groups to 9.0 in the 40+. These people had not been treated with antibiotics. From this era, approximately 75% of people known to be carriers have a history consistent with a clinical typhoid infection. With problems with diagnosis, and the general frequency of febrile disease in this era, it is not clear if the other 25% did not at some time have typhoid fever or if they genuinely became carriers following only a sub-clinical infection [16].

			The impact of antibiotic treatment on the probability of typhoid patients becoming carriers is not clear. In an outbreak in 1964, 507 people in Aberdeen were infected from contaminated corned beef and were treated with chloramphenicol. Of these, 5 are known to become carriers, despite treatment of many of the temporary carriers with ampicillin as a trial to reduce temporary and chronic carriage. On the other hand, in the published series of volunteer studies, none of the 250 people infected and sick enough to be treated with drugs (215 with chloramphenicol), nor any of volunteers with sub-clinical infections became chronic carriers [17].
P_4	Proportion of patients that become temporary Carriers/ had relapses	0.3	***Derived from literature values***<br>This model assumes that no hospital treated nor adequately treated community patients become temporary carriers since modern antibiotics should clear temporary carriage. For inadequately or untreated patients, the proportion of patients that continue to secrete after one month [10] is commonly quoted as 10% with an average time of approximately 4 months.<br>In the published results of the volunteer trials, 10% of volunteers that were not treated with chloramphenicol (i.e. had relatively mild typhoid) had relapses. Volunteers treated with chloramphenicol had a higher relapse rate (15 to 20%)[11]. A high rate of continued excretion was reported in a series of 374 patients infected in 1938 and 1939 (i.e. prior to use of chloramphenicol) quoted by Ames [12], the proportion was much higher with 31% overall still with positive stools at 4 weeks (24.9% in the <30 and 43.2% in the 30+). In this series there was a close fit with an exponential decay of positivity (half-life of 1.9 weeks in the <30 and 2.6 weeks in the 30+). In the series reported by Garbat [13], approximately 48 of 163 infections (30%) of patients became temporary carriers and the decay closely fitted an exponential decay with a half-life of 4.6 weeks.
P_5	Case fatality rate for clinical infections	Untreated: 0.1<br>Community treated: 0.02<br>Hospital treated: 0.005	***Derived from literature values***<br>Case fatality rates vary depending on the quality of treatment and geographical area [10]. CFR in the absence of the best practice hospital care can be estimated from historical values and can high. Supplementary table 3 lists 82 literature values over the period 1849 to 2004. The CFR was remarkably constant at an average of 10.1% from 1849 to 1944 then fell to approximately 1% from 1950 with the advent of effective antibiotics.
P_6	Probability a clinical infection induces sterile immunity	Default value 0.184	***From sensitivity analysis and consistent with literature on repeat infections***<br>Marmion [14] summarizes the data from late 19th and early 20th century typhoid studies in Europe where even after taking into account difficulties in diagnosis, it appears that multiple episodes, while not common, were detected.<br>Marmion studied two outbreaks of typhoid in British air force personnel in Egypt where two outbreaks occurred 5 months apart with different phage types. In this case, 84 of 657 men at risk developed typhoid fever in the first attack. In the second outbreak 235 of 688 at risk contracted the disease. In this second outbreak there were 384 exposed but not clinically infected in the first
P_7	Probability a clinical infection induces clinical immunity	Default value 0.184. Conditional on the infection not inducing	

		sterile immunity.	outbreak. 146/384 (38%) developed typhoid fever in the second outbreak. Of 250 not exposed to the first outbreak, but present in the second, 78 developed typhoid fever (attack rate 31%). Of the 54 that developed typhoid in the first attack and who were exposed in the second, 11 (20%) developed typhoid in the second attack.<br><br>In the case of experimental challenge, 25% of those previously symptomatic developed typhoid when challenged with the same dose 2 to 12 months later [11]. In another study by the same group, a comparative trial was done with naive and previously infected volunteers re-challenged with 10^5 Quales strain Typhi bacteria [15]. In this case 5/22 (23%) of the previously ill volunteers developed disease and 11/34 (30%) of naive volunteers.<br><br>Taken together, the available data suggests that prior infection can induce at least clinical immunity but this occurs are a relatively low frequency.<br><br>Although it has been postulated that repeated "mild" episodes can induce immunity in field conditions [16], there is little direct evidence of this. In fact, in the volunteer studies, especially at a low challenge dose many of the volunteers secreted bacteria for some weeks, unless they became ill, there was no change circulating antibody levels suggesting little impact on systemic immunity [6].<br><br>The default values listed correspond to an average of 3 subclinical or clinical infections to give immunity (See main text). These come from a detailed sensitivity analysis and have been chosen to give incidence and average age of infection predictions that match field data.
P_8	Probability a subclinical infection induces sterile immunity in a host with pre-existing infection induced clinical immunity	Default value 0.184	
P_9	Probability a subclinical infection induces sterile immunity in a host with no pre-existing infection induced immunity	Default value 0.184	
P_{10}	Probability that a clinical infection induces clinical immunity in a host with no pre-existing infection	Default value 0.184. Conditional on the infection not inducing sterile immunity.	
P_{11}	Probability vaccine induces clinical immunity in a host that has never been vaccinated. Conditional on not inducing sterile immunity		***Not used in this publication.***<br>Depends on the vaccine. The effects of vaccination depend on the specific characteristics of the vaccine. Vaccine efficacy trials with currently licensed [17–22] or experimental vaccines [23,24] cannot distinguish between sterile immunity and clinical immunity and thus measure the probability of either sterile or clinical immunity.<br>Default values and ranges trialed for individual vaccines will be included in future publications that model vaccine deployment.
P_{12}	Probability vaccine induces sterile immunity in a host that has never been vaccinated		
P_{13}	Probability vaccine induces clinical immunity in a		

	vaccinated, but non-immune host. Conditional on not inducing sterile immunity		
P_{14}	Probability vaccine induces sterile immunity in a vaccinated, but non-immune host		
P_{15}	Probability vaccine induces sterile immunity in a host with vaccine-induced clinical immunity		
$\overline{\tau}_r, \sigma_r^2$	Duration of refractory/non-exposed period	No default value.	***Determined by fitting age dependent incidence***<br>See text – values are dependent on the population modeled
τ_x	Duration of Infection-induced clinical Immunity	160 months.	***Sensitivity analyses of values ranging from zero to lifelong (1600 months).***<br>See text. 160 months used as default, however the sensitivity analysis excludes zero but a wide range of values is possible.
τ_z	Duration of infection-induced sterile immunity	800 months	***Sensitivity analyses of values ranging from zero to lifelong (1600 months)***<br>See text. A long duration sterile immunity is required to give age distributions and incidence rates seen in endemic communities however values ranging from about 400 months to lifelong are possible, depending on assumptions about duration of clinical immunity
τ_v	Duration of vaccine-induced clinical immunity		***Not used in this paper***<br>Depends on the vaccine. Field estimates of the length of vaccine induced immunity measure the sum of lengths of clinical and sterile immunity.<br>Default values and ranges trialed for individual vaccines will be included in future publications that model vaccine deployment.
τ_w	Duration of vaccine-induced sterile immunity		
τ_p	Duration of pre-patent period (months)	0.25	***Derived from literature values***<br>Measured pre-patent periods from volunteer studies [3] and the period inferred from outbreak studies [4] shows that the pre-patent period depends on the dose of infecting organisms. A geometric mean of 9.2 days was determined for low dose infections in volunteers [3]. Estimates of prepatent periods from epidemic data suggest a mean incubation time of 13.2 and 18.5 days with 16.7 and 3.8% attack rates estimated from food and water borne epidemics [4]. As shorter periods (4.5 days) were associated with very high inocula (10^8 to 10^9) that seem unlikely to be present in the environment, the longer estimate has been used in this modeling.<br>From volunteer studies, Typhi were present in the stool samples of most volunteers within 24 hours

			regardless or not of whether they became ill. In volunteers who became ill, stool samples were frequently negative during the febrile period, but becoming positive again following treatment with chloramphenicol [25]. In the detailed cases quoted, secretion of bacteria had ceased by one month. Hornick in summarizing his studies concluded that chloramphenicol "did not shorten the period of multiplication or residence of typhoid bacilli in the gut" [25].
τ_s	Duration of subclinical infections (months)	0.83	***Derived from literature values***<br>The very few observations of the duration of a subclinical infection come from volunteer studies. The limited data suggest a period similar to the duration of a clinical episode [25].<br>For simplicity of modeling, subclinical infections are nominally divided into a prepatent period, of similar duration as the prepatent period in clinical infections and a sub-clinical duration. In practice, the prepatent period and the subclinical duration are unlikely to be detected in field samples, but the duration of "prepatent" + "subclinical" are observable in volunteer studies.
τ_a	Duration of clinical infections(months)	0.83<br>0.5<br>0.25	***Derived from literature values with assumptions on speed of treatment***<br>For untreated or inadequately treated cases where the patient survives, estimates from the pre-antibiotic era suggested durations of approximately 25 days (0.83 months) after illness developed [13]. When patients are adequately treated or die, in this model it is assumed that the infection ceases on the day that treatment or death prevents further excretion of Salmonella into the environment.<br>In this model, we assume that cases severe enough to be hospitalized or that receive appropriate drugs in the community but still die will be infectious for 1 week (0.25 months), cases that are adequately treated in the community or are untreated and die will be infectious for 2 weeks (0.5 months).
τ_t	Duration of temporary carrier state	Half-life of 1 month	***Derived from literature values***<br>See comments above on proportion of infections that become temporary carriers
β_p	Infectiousness of prepatent infections	4 per month	***Estimated but relative levels consistent with literature. Only the relative infectiousness compared to other infection states, not the absolute value impacts model outcomes***<br>The limited data come from volunteer studies and are approximately proportional to the proportion of volunteers who were stool positive. This does not take into account the levels of excretion (bacteria per gram of stool), data which would be useful but is unavailable even for the volunteer challenge studies.
β_s	Infectiousness of subclinical infections	1.2 per month	
β_a	Infectiousness of clinical infections	4 per month	

β_c and β_t	Infectiousness of carrier states	1 per month of infection	***Estimated but relative levels consistent with literature. Only the relative infectiousness compared to other infection states, not the absolute value impacts model outcomes***<br>Some carriers only intermittently excrete bacteria [13] and when excreting, there is a wide range of excretion levels. In one series of 13 carriers from New York State, the range was a million fold from 10^4 to 10^{10} bacteria per gram of stool [26]. In longitudinal studies, while there is variation in the density of Typhi in successive stool samples from an individual, samples from one individual tend to be more consistent that samples from different carriers.<br>In the modeling presented in this paper, it is assumed that all carriers secrete approximately the same number of bacteria per month as an active case will in the entire episode. However as detailed in the text, the model allows carriers to be assigned to up to 10 different classes of infectiousness to accommodate the observed diversity of carrier infectiousness.

Table S2. Typhoid Incidence estimates in endemic sites

Country	Site	Age group observed	Age specific incidence in study population (per 100,000 per year)	Overall incidence in total population (per 100,000 per year)	Person years followed	comment	Surveillance	Case definition	Reference
South Africa	Eastern Transvaal	5-16	472		9961	Vaccinated with Pneumococcal PS	Active	Blood culture	Klugman 1987[21]
		5-16	846		20459	Non-vaccinated	Active	Blood Culture	
Chile	Area Occidente, Santiago	5-9	116		7193	3 year follow up. Age at start of study	passive	Culture (blood, bone marrow, duodenal fluid)	Levine 1987[27]
		10-14	110		9710				
		15-19	87		5001				
Egypt	Alexandria	5-9	138		15902		active	Culture or symptoms+Widal	Wahdan 1982[28]
Egypt	Fayoum	0-4	6		378316	Corrections for referral rates and sensitivity of tests applied	passive	Blood culture	Srikantiah 2006 [29]
		5-9	143		295040				
		10-14	160		280263				

		>=15	34		1284971				
		all ages		*59*					
Nigeria	Ilesha	All ages		18	About 200000	Predominantly young children. Peak 18 to 24 months	passive	Blood culture	Duggan 1975 [30]
Egypt	Alexandria	6-7	209		21017		active	Culture or symptoms+Widal	Wahdan 1975[31]
Pakistan	Hijrat Colony, Sultanabad & Bilal Colony, Karachi	All ages		405	101937	Survey done in 2003	Passive	Blood culture	Ochiai 2008[32]
India	Ward 29 & 30 Kolkata	All ages		495	56946				
Indonesia	Tanjung Priok & Koja, North Jakarta	All ages		190	160261				
Vietnam	Hue	All ages		20	281781				
China	Jin-Cheng Jiang & Dong Jiang, Hechi,	All ages		25	112889				

	Guangxi								
Pakistan	Coastal peri-urban Karachi	<2	443		1600		Active weekly visit	Blood culture	Owais 2010[33]
		<5	405		3950				
Pakistan	Sultanabad and Hijrat Colony, Karachi	<16	170				Active, every 2 weeks	Blood culture	Siddiqui 2006[1]
		<16	700					Serological + symptoms	
Vietnam	Cao Lanh District, Dong Thap Province, Mekong Delta	All ages		198			Passive	Blood culture	Lin 2000[2]
		5-9	581					Blood culture	
		All ages		2323				Clinical symptoms (includes blood culture positive	
India, Delhi	Geeta Colony, Delhi	1-15	726		6428		Active, every 4th day	Blood or stool culture + clinical symptoms	Chuttani 1973[34]
		6-17	1270		5104				
India	Narkeldanga, Kolkata	All ages		370		In high risk areas. Peak rate age 10-19	Passive	Blood culture	Sur 2006[35]
				80		Low risk areas			
		10-19	500			Age distribution			
Bangladesh,	Kamalapur,			200	24893		Active,	Blood culture	Naheed

	Dhaka	<5	1050			Under 5 subset	weekly visit		2010[36]
India	Kalkaji, New Delhi	<5	2730		1027	migrants	Active, twice per week	Blood culture	Sinha 1999[37]
		5-19	1170		2743				
		10-40	11		2684				
		<40	980						
India	Kurseong sub-division of Darjeeling district	>5	5	28	19287		Passive	Fever for 3 days + 4 fold rise in anti-O	Sharma 2009[38]
		5-14	28		45737				
		15-29	20		54003				
		30+	18		64657				
Nepal, Kathmandu	Lalitpur Sub-<br>Metropolitan City, Kathmandu,	All ages		59 (range in subgroups 7 to 100)	162997	Substantial migrant component	Passive	Positive blood culture	Karkey 2010[39]
India Delhi	Geeta Colony, Delhi	1 -17	1274		6248	Adjusted to 12 months	Active. Visit every 4 days	Blood culture or stool culture + clinical symptoms	Chuttani 1971[40]
Nepal	five villages west of Kathmandu	5-40	1166		4888	Vaccine had same efficacy on symptoms only as on blood	Active. Visit every 2 days	Blood culture positive + culture negative with symptoms	Acharya 1987[41]

		5-40	664		4888	positive only.		Blood culture positive only	

Table S3. Case fatality rates

Study	Years	case fatality rate %	No. of deaths	Comments	References
Review of 61 outbreaks	1881-1944	10.0%	2,000	10% consistent 1881 -1944 then marked reduction 1950+ outbreaks	[4]
Review of 6 outbreaks	1950-1971	0.76%	6		
References not included in [4]					
Bath UK	1849	6%	40		[42]
Massachusetts Dept of Health	1875-1934	10%	28,657		[43]
USA Army	1898	7.6%	1,580	20,738 infected out of 107,933 personnel	[44]
Sheboygan, Wisconsin	1906-1908	12%	21		[45]
USA Navy	1890-1911	8.9%	252	Pre-vaccine	[46]
USA Navy	1912-1933	7.7%	44	Post vaccination	
Community conservation corps	1933	7.4%	4	All vaccinated	[47]
UK airmen Shallufa Egypt	1945	10%	11	14% attack rate	[48]
Data used for modelling	1971	3%		Range 1% to 10%	[49]
Goroka, PNG	1984-1990	10-15%		44% CFR in a subgroup	[50]

Aga Khan University, Medical Center, Karachi,	1988-1993	1.6%	19	Higher death rate in children	[51]
International Centre for Diarrhoeal Disease Research, Bangladesh	1990-1993	4.2%	14		[52]
Hospitalized Sth Vietnam	1993-1994	0.3%			[53]
Review	2002	1%		Average of all patients, 2-50% in hospitalized	[10]
Congo	2004	44%	64	Peritonitis patients	[54]

References

1. Siddiqui FJ, Rabbani F, Hasan R, Nizami SQ, Bhutta ZA (2006) Typhoid fever in children: some epidemiological considerations from Karachi, Pakistan. Int J Infect Dis 10: 215-222.

2. Lin FY, Vo AH, Phan VB, Nguyen TT, Bryla D, Tran CT, Ha BK, Dang DT, Robbins JB (2000) The epidemiology of typhoid fever in the Dong Thap Province, Mekong Delta region of Vietnam. Am J Trop Med Hyg 62: 644-648.

3. Glynn JR, Hornick RB, Levine MM, Bradley DJ (1995) Infecting dose and severity of typhoid: analysis of volunteer data and examination of the influence of the definition of illness used. Epidemiol Infect 115: 23-30.

4. Glynn JR, Bradley DJ (1992) The relationship between infecting dose and severity of disease in reported outbreaks of Salmonella infections. Epidemiol Infect 109: 371-388.

5. Hornick RB (1970) Pathogenesis of typhoid fever. J Egypt Public Health Assoc 45: 247-259.

6. Hornick RB, Woodward TE (1967) Appraisal of typhoid vaccine in experimentally infected human subjects. Trans Am Clin Climatol Assoc 78: 70-78.

7. Glynn JR, Palmer SR (1992) Incubation period, severity of disease, and infecting dose: evidence from a Salmonella outbreak. Am J Epidemiol 136: 1369-1377.

8. Stebbins E.L., Reed E. (1937) Carrier-Borne Typhoid Fever in New York State: With Special Reference to Attack Rates Among Household Contacts. Am J Public Health Nations Health 27: 233-240.

9. Levine MM, Black RE, Lanata C (1982) Precise estimation of the numbers of chronic carriers of Salmonella typhi in Santiago, Chile, an endemic area. J Infect Dis 146: 724-726.

10. Parry CM, Hien TT, Dougan G, White NJ, Farrar JJ (2002) Typhoid fever. N Engl J Med 347: 1770-1782.

11. Hornick RB, Greisman SE, Woodward TE, Dupont HL, Dawkins AT, Snyder MJ (1970) Typhoid fever: pathogenesis and immunologic control. 2. N Engl J Med 283: 739-746.

12. Ames WR, Robins M (Age and Sex as Factors in the Development of the Typhoid Carrier State, and a Method for Estimating Carrier Prevalence. 33: 221-230.

13. Garbat, A. (1922) Typhoid carriers and typhoid immunity. New York: The Rockefeller Institute for Medical Research. 110 p.

14. Marmion DE, Naylor GR, Stewart IO (1953) Second attacks of typhoid fever. J Hyg (Lond) 51: 260-267.

15. Dupont HL, Hornick RB, Snyder MJ, Dawkins AT, Heiner GG, Woodward TE (1971) Studies of immunity in typhoid fever. Protection induced by killed oral antigens or by primary infection. Bull World Health Organ 44: 667-672.

16. Ashcroft MT (1964) Typhoid and paratyphoid fevers in the tropics. J Trop Med Hyg 67: 185-189.

17. Levine MM, Ferreccio C, Abrego P, Martin OS, Ortiz E, Cryz S (1999) Duration of efficacy of Ty21a, attenuated Salmonella typhi live oral vaccine. Vaccine 17 Suppl 2: S22-S27.

18. Levine MM, Ferreccio C, Cryz S, Ortiz E (1990) Comparison of enteric-coated capsules and liquid formulation of Ty21a typhoid vaccine in randomised controlled field trial. Lancet 336: 891-894.

19. Wahdan MH, Serie C, Germanier R, Lackany A, Cerisier Y, Guerin N, Sallam S, Geoffroy P, el Tantawi AS, Guesry P (1980) A controlled field trial of liver oral typhoid vaccine Ty21a. Bull World Health Organ 58: 469-474.

20. Klugman KP, Koornhof HJ, Robbins JB, Le Cam NN (1996) Immunogenicity, efficacy and serological correlate of protection of Salmonella typhi Vi capsular polysaccharide vaccine three years after immunization. Vaccine 14: 435-438.

21. Klugman KP, Gilbertson IT, Koornhof HJ, Robbins JB, Schneerson R, Schulz D, Cadoz M, Armand J (1987) Protective activity of Vi capsular polysaccharide vaccine against typhoid fever. Lancet 2: 1165-1169.

22. Fraser A, Goldberg E, Acosta CJ, Paul M, Leibovici L (2007) Vaccines for preventing typhoid fever. Cochrane Database Syst Rev CD001261.

23. Lin FY, Ho VA, Khiem HB, Trach DD, Bay PV, Thanh TC, Kossaczka Z, Bryla DA, Shiloach J, Robbins JB, Schneerson R, Szu SC (2001) The efficacy of a Salmonella typhi Vi conjugate vaccine in two-to-five-year-old children. N Engl J Med 344: 1263-1269.

24. Mai NL, Phan VB, Vo AH, Tran CT, Lin FY, Bryla DA, Chu C, Schiloach J, Robbins JB, Schneerson R, Szu SC (2003) Persistent efficacy of Vi conjugate vaccine against typhoid fever in young children. N Engl J Med 349: 1390-1391.

25. Hornick RB, Greisman SE, Woodward TE, Dupont HL, Dawkins AT, Snyder MJ (1970) Typhoid fever: pathogenesis and immunologic control. N Engl J Med 283: 686-691.

26. Merselis JG, Jr., KAYE D, Connolly CS, Hook EW (1964) Quantitative bacteriology of the typhoid carrier state. Am J Trop Med Hyg 13: 425-429.

27. Levine MM, Ferreccio C, Black RE, Germanier R (1987) Large-scale field trial of Ty21a live oral typhoid vaccine in enteric-coated capsule formulation. Lancet 1: 1049-1052.

28. Wahdan MH, Serie C, Cerisier Y, Sallam S, Germanier R (1982) A controlled field trial of live Salmonella typhi strain Ty 21a oral vaccine against typhoid: three-year results. J Infect Dis 145: 292-295.

29. Srikantiah P, Girgis FY, Luby SP, Jennings G, Wasfy MO, Crump JA, Hoekstra RM, Anwer M, Mahoney FJ (2006) Population-based surveillance of typhoid fever in Egypt. Am J Trop Med Hyg 74: 114-119.

30. Duggan MB, Beyer L (1975) Enteric fever in young Yoruba children. Arch Dis Child 50: 67-71.

31. Wahdan MH, Sippel JE, Mikhail IA, Rahka AE, Anderson ES, Sparks HA, Cvjetanovic B (1975) Controlled field trial of a typhoid vaccine prepared with a nonmotile mutant of Salmonella typhi Ty2. Bull World Health Organ 52: 69-73.

32. Ochiai RL, Acosta CJ, Danovaro-Holliday MC, Baiqing D, Bhattacharya SK, Agtini MD, Bhutta ZA, Canh dG, Ali M, Shin S, Wain J, Page AL, Albert MJ, Farrar J, Abu-Elyazeed R, Pang T, Galindo CM, von SL, Clemens JD (2008) A study of typhoid fever in five Asian countries: disease burden and implications for controls. Bull World Health Organ 86: 260-268.

33. Owais A, Sultana S, Zaman U, Rizvi A, Zaidi AK (2010) Incidence of typhoid bacteremia in infants and young children in southern coastal Pakistan. Pediatr Infect Dis J 29: 1035-1039.

34. Chuttani CS, Prakash K, Vergese A, Gupta P, Chawla RK, Grover V, Agarwal DS (1973) Ineffectiveness of an oral killed typhoid vaccine in a field trial. Bull World Health Organ 48: 756-757.

35. Sur D, von SL, Manna B, Dutta S, Deb AK, Sarkar BL, Kanungo S, Deen JL, Ali M, Kim DR, Gupta VK, Ochiai RL, Tsuzuki A, Acosta CJ, Clemens JD, Bhattacharya SK (2006) The malaria and typhoid fever burden in the slums of Kolkata, India: data from a prospective community-based study. Trans R Soc Trop Med Hyg 100: 725-733.

36. Naheed A, Ram PK, Brooks WA, Hossain MA, Parsons MB, Talukder KA, Mintz E, Luby S, Breiman RF (2010) Burden of typhoid and paratyphoid fever in a densely populated urban community, Dhaka, Bangladesh. Int J Infect Dis 14 Suppl 3: e93-e99.

37. Sinha A, Sazawal S, Lackany A, Sood S, Reddaiah VP, Singh B, Rao M, Naficy A, Combs BG, Bhatta DR (1999) Typhoid fever in children aged less than 5 years. Lancet 354: 734-737.

38. Sharma PK, Ramakrishnan R, Hutin Y, Manickam P, Gupte MD (2009) Risk factors for typhoid in Darjeeling, West Bengal, India: evidence for practical action. Trop Med Int Health 14: 696-702.

39. Karkey A, Arjyal A, Anders KL, Boni MF, Dongol S, Koirala S, My PV, Nga TV, Clements AC, Holt KE, Duy PT, Day JN, Campbell JI, Dougan G, Dolecek C, Farrar J, Basnyat B, Baker S (2010) The burden and characteristics of enteric fever at a healthcare facility in a densely populated area of Kathmandu. PLoS One 5: e13988.

40. Chuttani CS, Vergese A, Sharma U, Singha P, Ray BG (1971) Effectiveness of oral killed typhoid vaccine. Bull World Health Organ 45: 445-450.

41. Acharya IL, Lowe CU, Thapa R, Gurubacharya VL, Shrestha MB, Cadoz M, Schulz D, Armand J, Bryla DA, Trollfors B (1987) Prevention of typhoid fever in Nepal with the Vi capsular polysaccharide of Salmonella typhi. A preliminary report. N Engl J Med 317: 1101-1104.

42. Davies W (1850) An Account of an Epidemic of Typhoid Fever, Which Prevailed in Bath: During the Months of September and October, 1849. Prov Med Surg J 14: 60-65.

43. Anderson GW, Hamblen AD, Smith HM (1936) Typhoid Carriers -A Study of Their Disease Producing Potentialities Over a Series of Years as Indicated by a Study of Cases. Am J Public Health Nations Health 26: 396-405.

44. Anon (1902) The Report on the Typhoid Fever Epidemics in the Volunteer Camps of the United States Army in 1898. Br Med J 2: 263-265.

45. Bowles JT (1909) Investigation of Typhoid Fever Epidemic at Sheboygan, Wisconsin. Am J Public Hygiene 19: 268-273.

46. Cook SS (1935) Efficacy of Typhoid Prophylaxis in the United States Navy. Am J Public Health Nations Health 25: 251-257.

47. Lull GF (1934) Fevers of the Typhoid Group in Members of the Civilian Conservation Corps During 1933. Am J Public Health Nations Health 24: 631-632.

48. Anderson ES, Richards HG (1948) An outbreak of typhoid fever in the Middle East. J Hyg (Lond) 46: 164-172.

49. Cvjetanovic B, Grab B, Uemura K (1971) Epidemiological model of typhoid fever and its use in the planning and evaluation of antityphoid immunization and sanitation programmes. Bull World Health Organ 45: 53-75.

50. Richens J (1995) Typhoid in the highlands of Papua New Guinea 1984-1990: a hospital-based perspective. P N G Med J 38: 305-314.

51. Bhutta ZA (1996) Impact of age and drug resistance on mortality in typhoid fever. Arch Dis Child 75: 214-217.

52. Azad AK, Islam R (1997) Comparison of clinical features and pathologic findings in fatal cases of typhoid fever during the initial and later stages of the disease. Am J Trop Med Hyg 56: 490-493.

53. Hoa NT, Diep TS, Wain J, Parry CM, Hien TT, Smith MD, Walsh AL, White NJ (1998) Community-acquired septicaemia in southern Viet Nam: the importance of multidrug-resistant Salmonella typhi. Trans R Soc Trop Med Hyg 92: 503-508.

54. Muyembe-Tamfum JJ, Veyi J, Kaswa M, Lunguya O, Verhaegen J, Boelaert M (2009) An outbreak of peritonitis caused by multidrug-resistant Salmonella Typhi in Kinshasa, Democratic Republic of Congo. Travel Med Infect Dis 7: 40-43.
