# Supplementary material for: Stochastic Simulation of Endemic Salmonella enterica Serovar Typhi: The Importance of Long Lasting Immunity and the Carrier State
Source: PLoS One. 2013 Sep 10;8(9):e74097. doi: 10.1371/journal.pone.0074097 (PMC3769365; doi:10.1371/journal.pone.0074097)
Supplement: File S2 — Description of software, input and output files. (DOCX) [file pone.0074097.s002.docx]

## File S2 Software Implementation

The model is implemented in Delphi 2005 (Borland Software Corporation, www.borland.com) and compiled to run on Windows and Linux based machines. The program uses a control file to define the model being examined with the following files to define parameters required to run the model. All time and time related probability parameters are expressed in months or per month, respectively

Survival file: Age specific probability of surviving each per month

Immune matrix file: Age specific immune transition probabilities following infection and vaccination, and the distribution of duration for the different immune states.

Infectiousness file: age and strain specific infectiousness rate for each type and stage of infection. Since chronic carriers have major diversity, the model allows chronic carriers to be assigned to up to 10 different classes of chronic carrier, each with their own age and strain specific infectiousness rate.

Outcome probability file: Age and strain specific probabilities of developing the different types of clinical outcome on infection (e.g. sub-clinical, clinical treated in hospital, typhoid death); the age, gender and strain specific probabilities of developing temporary and chronic carrier status; the probability of being in each of the 10 possible classes of the population and the probability of being reassigned from one class to another as a result of an intervention; and the age and strain specific exposure and infectivity within each population class.

Migration file (optional): If present, specificities the average in-migration rate, the duration of migration, and the average age distribution, gender, infection and immune status of migrants.

In each time step, using Bernoulli sampling from with a random number generator, the program calculates the

1. Number of births
2. Who dies from causes other than typhoid
3. Who becomes infected and who will develop sub-clinical or clinical infections following the prepatent period.
4. Who dies from typhoid
5. Who is treated
6. Who becomes a temporary carrier and who clears a temporary carriage state
7. Who becomes a lifelong carrier and their level of infectiousness
8. Who gains natural immunity, either sterile or clinical, and the corresponding duration of immunity
9. Who loses natural immunity (transition from sterile to clinical or complete loss of immunity)
10. Who is vaccinated, become immune (sterile or clinical) and the duration of the immunity.
11. Who loses vaccine induced immunity
12. The number of people migrating into the community (and their individual status with regard to infection, immunity age and gender)

The time step is specified by the investigator as an integral fraction of a month (typically ¼ or approximately one week). A range of values from one day (1/30 month) to a maximum of 1 month has been evaluated.

The main disease output of the model are the number and age distribution of typhoid “cases”, i.e. clinically ill patients, as a function of time. Other outputs include

1. the number of non-typhoid deaths,
2. number of treatments as a function of age,
3. number of vaccinations as a function of age,
4. number of carriers,
5. population size and age distribution,
6. number and age distribution of people with infection induced clinical or sterile immunity
7. number and age distribution of people with vaccine induced clinical or sterile immunity
8. age specific cases in vaccinated and non-vaccinated individual to allow calculation of efficacy and herd protection
